# Performance of ChatGPT-4o in Providing Information on Pediatric Inborn Errors of Immunity: A Cross-Sectional Evaluation

**DOI:** 10.3390/jcm15114025

**Published:** 2026-05-22

**Authors:** İlke Taşkırdı, Ece Şenbaykal Yiğit, Sanem Eren Akarcan, Tuba Tuncel

**Affiliations:** 1 Department of Pediatrics, Division of Pediatric Allergy and Immunology, Faculty of Medicine, Izmir Katip Celebi University, Izmir 35620, Turkey; ttuncel@yahoo.com.tr; 2 Department of Pediatrics, Division of Pediatric Allergy and Immunology, Izmir City Hospital, Izmir 35540, Turkey; ecesenbaykal@hotmail.com (E.Ş.Y.); saneren@yahoo.com (S.E.A.)

**Keywords:** ChatGPT, artificial intelligence, inborn errors of immunity, pediatric, patient education, readability, health literacy

## Abstract

**Background/Objectives:** Inborn errors of immunity (IEI) are rare and complex pediatric disorders that create significant information gaps for families and non-specialist healthcare professionals. Large language models (LLMs) such as ChatGPT are increasingly used as on-demand health information resources; however, evidence on their performance in rare pediatric diseases remains limited. This study aimed to evaluate the reliability, quality, readability, understandability, reproducibility, and safety-related concerns of ChatGPT-4o responses to frequently searched questions about pediatric IEI posed by healthcare professionals and patients/caregivers. **Methods:** This cross-sectional evaluation used the publicly accessible ChatGPT-4o interface to generate responses to 20 frequently searched questions about pediatric IEI, equally distributed between healthcare professional (n = 10) and patient/caregiver queries (n = 10). Three pediatric allergy-immunology specialists independently evaluated response quality using the modified DISCERN (mDISCERN) and Global Quality Scale (GQS) tools, supplemented by a structured expert-based assessment of misinformation, safety-related concerns, suspected factual issues, missing disclaimers, and clinically meaningful inter-iteration inconsistency. Text readability was assessed using four validated indices (ARI, FRES, FKGL, GFR), comprehensibility using the Patient Education Materials Assessment Tool (PEMAT), and reproducibility using natural language processing methods. **Results:** ChatGPT-4o demonstrated strong overall performance, with median mDISCERN and GQS scores of 4 (IQR: 3–5) for both query types. Readability scores substantially exceeded recommended thresholds, with FKGL scores of 12.96 ± 0.69 and 10.83 ± 0.67 for professional and patient/caregiver queries, respectively. Mean PEMAT understandability scores were 71.80 ± 5.75% for professional queries and 80.80 ± 4.73% for patient/caregiver queries (*p* = 0.001). Reproducibility was high, with semantic similarity rates of 86.10 ± 3.84% and 87.30 ± 3.68%, respectively. Suspected factual issues were identified in 4 of 20 responses (20%), safety-related concerns in 3 (15%), clinically meaningful inter-iteration inconsistencies in 3 (15%), and missing medical disclaimers in all 20 responses (100%). **Conclusions:** ChatGPT-4o showed strong performance across validated quality metrics for pediatric IEI information support; however, its high reading level, universal absence of medical disclaimers, and occasional clinically meaningful inconsistencies limit its suitability as a standalone source for clinically sensitive guidance. These findings underscore the need for AI-driven patient education tools with improved readability, adaptive complexity adjustment, and safety-oriented communication.

## 1. Introduction

Inborn errors of immunity (IEI) are a heterogeneous group of rare disorders caused by defects in immune system development or function, often presenting with recurrent infections, immune dysregulation, autoinflammation, allergy, or malignancy [1,2,3,4,5]. Their clinical manifestations are highly variable, and diagnosis, prognosis, and management frequently require specialized expertise. This inherent complexity, coupled with the rarity of these conditions, creates a substantial information gap for families and non-specialist healthcare professionals, who may struggle to access timely, specialized guidance outside tertiary referral centers. Consequently, there is an increasing shift toward digital resources to bridge these knowledge gaps in real time.

Large language models (LLMs) are artificial intelligence systems capable of generating human-like text in response to user prompts and are increasingly used for question answering, summarization, and health information support [6,7]. Among publicly available LLMs, ChatGPT has emerged as a prominent and widely accessible application. However, the linguistic fluency of these models does not inherently guarantee clinical adequacy; moreover, out-of-the-box performance in typical user-facing interfaces may differ substantially from outcomes achieved through specialized prompt engineering or fine-tuning.

Interest in the use of LLMs in healthcare has grown rapidly because these tools can provide immediate, on-demand responses to health-related questions. This may be particularly appealing in rare and complex diseases such as IEI, where families may search for understandable explanations and frontline healthcare professionals may seek concise background information. However, it is critical to emphasize that user preference for chatbot-generated responses—often driven by their perceived empathy and fluency—does not necessarily equate to factual accuracy, safety, or clinical appropriateness [8]. Recent pediatric studies have reinforced this distinction by showing that chatbot responses perceived as clear, useful, or trustworthy by users may still contain clinically important limitations when evaluated by specialists, particularly with respect to safety, completeness, readability, or treatment-related adequacy [9,10,11,12]. In specialized pediatric conditions, where communication must be both nuanced and evidence-based, overreliance on the conversational quality of AI may mask underlying risks.

IEI pose distinctive challenges for AI-generated content because of their rarity, which limits general familiarity, and their phenotypic heterogeneity, which complicates generalization. In pediatric immunology, where prognostic uncertainty is common, there is effectively zero tolerance for inconsistent or overly simplified messaging. Given that counseling in IEI involves high-stakes decisions regarding long-term prognosis, infection precautions, and complex treatment regimens, even minor inaccuracies or clinically meaningful inter-iteration inconsistencies can have profound consequences for family decision-making and patient safety. These features make pediatric IEI a particularly rigorous test case for evaluating whether LLMs can provide information that is not only fluent but also clinically responsible and safe.

Although an expanding body of literature has evaluated LLM performance in more common medical conditions, evidence in rare pediatric diseases remains comparatively sparse, largely limited to disease-specific or pilot-level studies in areas such as rare pediatric case diagnosis, genetic counseling support, Dravet syndrome, and off-label prescribing [13,14,15,16,17]. Existing studies in child health have raised recurring concerns regarding readability, source transparency, treatment-related completeness, safety, and audience adaptation, suggesting that apparently fluent responses may still harbor important limitations even when overall quality or user-perceived usefulness appears favorable [9,10,11,12,18,19]. In rare diseases such as IEI, these limitations may be especially consequential because patients and caregivers often rely on scarce and fragmented information sources, while non-specialist clinicians may also need accessible decision support.

Within this rapidly expanding evidence base, the present study seeks to contribute in three specific ways. First, to our knowledge it represents the first dedicated, multidimensional evaluation of LLM performance in pediatric IEI, a rare-disease area in which the combination of long-term prognostic uncertainty, infection-prevention complexity, and advanced therapeutic considerations (including hematopoietic stem cell transplantation and emerging gene therapies) makes information adequacy especially consequential. Second, by integrating validated quality instruments (mDISCERN, GQS), four readability indices, the PEMAT, NLP-based reproducibility analysis, and a structured expert-based safety screen within a single framework, this study offers a methodological template that can be transferred to other rare pediatric conditions. Third, by stratifying analyses by intended audience (healthcare professional versus patient/caregiver), we examine whether—and how reliably—a public-facing LLM adapts content to user role, a feature central to safe deployment in specialty pediatric care.

This study aimed to evaluate the publicly accessible, out-of-the-box performance of ChatGPT-4o in responding to frequently searched questions about pediatric IEI. We assessed responses directed at both patients/caregivers and healthcare professionals to examine how the model performed across different user groups and to evaluate its audience-adaptation capabilities, a critical factor for safety in specialized pediatric care. Specifically, we evaluated reliability and quality using validated tools, readability and comprehensibility using established text assessment instruments, reproducibility across repeated queries, and supplementary expert-based indicators of misinformation and safety-related concerns. We sought to determine whether ChatGPT-4o can provide information that is not only fluent and high-quality in appearance but also appropriately understandable, consistent, and clinically responsible in the context of pediatric IEI.

## 2. Methods

### 2.1. Study Design

This cross-sectional evaluation was conducted between late May and July 2025, following Institutional Review Board approval issued on 15 May 2025, using the publicly accessible ChatGPT-4o interface (https://chatgpt.com). The publicly accessible version was selected to reflect typical user access patterns, particularly those of patients, caregivers, and non-specialist healthcare professionals who are more likely to use widely available AI tools in routine information-seeking contexts. Because large language models evolve rapidly, this design was intended to evaluate ChatGPT-4o performance at a defined time point under real-world access conditions. Comparative benchmarking against alternative LLMs, curated clinical decision support resources, or established patient-education repositories was deliberately deferred to a follow-up study, so that the present evaluation could provide an in-depth, multidimensional baseline characterization of a single, widely used public-facing model under conditions reflecting typical end-user access.

### 2.2. Question Selection and Data Collection

Twenty frequently searched questions regarding pediatric IEI were identified using a structured search-analytics procedure based on publicly available Google tools, conducted in May 2025 following Institutional Review Board approval. Specifically, we combined three complementary inputs: (i) Google Trends queries (worldwide, English-language, 12-month window) for the seed terms “primary immunodeficiency,” “inborn errors of immunity,” “PID children,” and “immune deficiency child”; (ii) Google Search autocomplete suggestions generated when the same seed terms were entered into the default search interface; and (iii) “People also ask” and “Related searches” expansions returned for the same seed queries. The aggregated candidate questions were screened by two of the authors (İ.T. and E.Ş.Y., both pediatric allergy–immunology specialists) for thematic relevance to pediatric IEI and removed if duplicated, off-topic, or unanswerable in a single-turn prompt. Candidate items were then categorized by anticipated audience (healthcare professional versus patient/caregiver) on the basis of vocabulary, clinical specificity, and information depth. Final selection was reached by consensus between the two screeners; disagreements were resolved through discussion with the senior author (T.T.). The final dataset consisted of 20 questions equally distributed between healthcare professional queries (n = 10) and patient/caregiver inquiries (n = 10) to reflect both major real-world user groups.

Each question was submitted to the AI system three times on different days and from different devices to assess response consistency and minimize session-dependent bias. All interactions used standardized single-turn prompts without additional context, follow-up questions, prompt engineering, or fine-tuning to preserve methodological consistency and reflect typical out-of-the-box use. All queries were submitted through the default public interface settings, without user control over internal parameters such as temperature or randomness. Responses were systematically documented and prepared for subsequent analysis. All questions and responses are available in the Supplementary Materials.

### 2.3. Evaluator Selection

Three pediatric allergy–immunology specialists with clinical experience in the diagnosis and management of pediatric IEI independently evaluated the responses through a comprehensive review process. Evaluator eligibility criteria included active clinical practice in pediatric allergy/immunology, experience with IEI-related patient care, and academic familiarity with the subject area. To ensure consistency, all reviewers used the same predefined assessment instruments and applied shared scoring criteria based on the definitions provided by each tool. The high inter-rater agreement observed across the principal quality measures further supported the consistency of the evaluation process.

### 2.4. Quality Assessment

Response quality and reliability were independently evaluated by three pediatric allergy–immunology specialists using two validated instruments: the modified DISCERN (mDISCERN) tool and the Global Quality Scale (GQS) [20,21,22]. The mDISCERN is a structured 5-point instrument that assesses key dimensions of health information quality, including clarity, reliability, balance, source attribution, and acknowledgment of uncertainty. The GQS provides an overall rating of information quality, flow, and usefulness on a 5-point scale, with higher scores indicating better overall quality.

These tools were selected to provide both domain-specific and overall assessments of AI-generated medical information. When interpreting findings, particular attention was paid not only to overall scores but also to domains such as source attribution and uncertainty acknowledgment, which are especially relevant when evaluating medical information provided to patients, caregivers, and non-specialist healthcare professionals.

We selected the mDISCERN, GQS, and PEMAT instruments because they remain the most widely applied tools in the contemporary AI-health information literature, providing direct comparability with prior LLM evaluation studies across multiple specialties [11,12]. We explicitly acknowledge that these instruments were originally developed and validated for human-authored, static consumer health information rather than for dynamic conversational AI outputs, and that no validated instrument tailored specifically to LLM-generated medical content is currently available. To partially mitigate this limitation, we supplemented the validated tools with the structured expert-based misinformation and safety assessment described below; the development of AI-specific quality instruments represents an important methodological priority for the field and is discussed further in the Limitations section.

### 2.5. Reference Standard for Accuracy Evaluation

Because no single externally curated benchmark exists for evaluating AI-generated content in pediatric IEI, we predefined a composite reference framework against which response accuracy and clinical appropriateness were judged. This framework included (i) the 2024 IUIS phenotypic classification of human inborn errors of immunity [1]; (ii) European Society for Immunodeficiencies (ESID) registry working definitions and consensus statements; (iii) widely cited pediatric immunology textbooks and recent peer-reviewed reviews [2,5]; and (iv) the clinical judgment of three board-certified pediatric allergy–immunology specialists with active practice in IEI. Each response was assessed against this composite framework, and discrepancies between the AI output and the framework formed the basis for the supplementary expert-based assessment described below. We acknowledge that this framework constitutes an expert-consensus standard rather than a formal head-to-head verification against a single regulatory benchmark; this design choice was made to preserve clinical realism in a rare-disease context where multiple complementary references guide everyday practice.

### 2.6. Supplementary Misinformation and Safety-Related Assessment

To complement the validated quality measures, we performed a structured expert-based supplementary assessment of misinformation and safety-related concerns, informed by the framework described by Alshak et al. [23]. Three pediatric allergy-immunology specialists independently reviewed each question–response set and evaluated the misinformation score, potential safety issues, suspected factual issues, missing medical disclaimers, and clinically meaningful inter-iteration inconsistency.

The presence of misinformation within the generated responses was scored on a standardized 5-point scale adapted from Alshak et al. [23]: 1 indicated none and 5 high; levels 2–4 represented minimal, moderate, and significant, respectively. Safety issues were operationally defined as content elements with the potential to (a) delay appropriate medical evaluation or care-seeking; (b) promote inappropriate self-management or modification of therapy; (c) generate false reassurance regarding clinically significant symptoms; (d) misrepresent the severity, prognosis, or treatment of IEI in a manner inconsistent with current pediatric IEI standards of care; or (e) provide age-inappropriate guidance for the pediatric population. Each candidate safety issue was first flagged independently by each evaluator using these criteria; an item was classified as a confirmed safety issue only if at least two of the three evaluators independently endorsed it under one or more of the above criteria. This pre-specified consensus rule was adopted to reduce subjectivity inherent in expert screening. Suspected factual issues were categorized according to the apparent domain of concern, including prognostic, precaution-related, and therapeutic issues. A missing disclaimer was defined as the absence of advice to consult a healthcare professional or equivalent safety-oriented language. Inter-iteration inconsistency was defined as clinically meaningful variation across the three repeated responses to the same question.

This supplementary assessment was designed as expert-based screening rather than formal independent verification of each medical statement against contemporary clinical guidelines or external reference standards. Accordingly, suspected factual issues should be interpreted as expert-identified concerns rather than confirmed errors, and safety issues as potential risk indicators rather than validated patient safety outcomes.

### 2.7. Readability Assessment

Text readability was evaluated using four validated indices: the Automated Readability Index (ARI), Flesch Reading Ease Score (FRES), Flesch-Kincaid Grade Level (FKGL), and Gunning Fog Readability Index (GFR) [24,25,26,27]. Readability formulas are provided in the Supplementary Materials. The ARI estimates the U.S. grade level required to comprehend a text based on characters per word and words per sentence. The FRES provides a score ranging from 0 to 100, with higher values indicating easier readability. The FKGL estimates the U.S. school grade level needed to understand the text. The GFR estimates the years of formal education required to understand a passage on first reading.

Readability was assessed for both patient/caregiver and healthcare professional responses. Although readability metrics are primarily designed for patient-facing educational materials, we also evaluated professional queries to examine whether the model adjusted linguistic complexity according to the intended audience. This distinction was considered relevant because not all healthcare professionals seeking information about pediatric IEI are subspecialists in immunology, and excessively complex responses may reduce practical usability even in professional contexts.

These formula-based indices provide standardized estimates of structural reading difficulty based on surface features such as sentence length and syllable count; they do not capture conceptual density, prior health literacy, familiarity with disease-specific terminology, or the unavoidable use of polysyllabic clinical vocabulary intrinsic to immunology. Accordingly, readability scores in this study should be interpreted as indicators of structural textual complexity rather than as direct measures of comprehension, and apparent failures to meet recommended thresholds may partly reflect the inherent linguistic demands of pediatric IEI as a topic rather than ineffective communication on the part of the model. The PEMAT understandability subscale and our reproducibility and expert-based assessments were therefore retained as complementary, content-aware indicators.

### 2.8. Comprehensibility Analysis

Comprehensibility was assessed using the Patient Education Materials Assessment Tool (PEMAT), a validated instrument developed to evaluate patient education materials [28]. The PEMAT generates percentage scores for two related but distinct domains: understandability, reflecting how easily users can process and explain key messages, and actionability, reflecting whether users can identify concrete actions based on the information provided.

In this study, understandability was considered central to evaluating information accessibility. Actionability was included as a complementary domain because effective health information should not only be understandable but also equip users with the capacity to recognize appropriate next steps when relevant. This distinction is particularly important for patient/caregiver queries, where practical guidance may influence health-related decision-making within the pediatric population.

Because the PEMAT was originally designed for patient-facing educational materials, its application to healthcare professional queries should be interpreted with caution. Accordingly, PEMAT findings in the professional group were used primarily to explore relative comprehensibility and usability rather than to make definitive judgments about the adequacy of professional-oriented educational content.

### 2.9. Reproducibility Assessment

To evaluate response consistency, each question was submitted to the publicly accessible ChatGPT-4o interface three times on different days and from different devices, with the aim of reducing session-dependent bias and capturing variation across repeated interactions under routine user conditions. The resulting responses were compared using natural language processing methods in Python 3 (version 3.11.4) and RStudio (version 2023.06.1+524).

Response similarity was assessed using cosine similarity and text classification approaches after standard preprocessing steps, including removal of stop words and consideration of semantic equivalence. Cosine similarity was selected because it measures semantic overlap while allowing for lexical variation and paraphrasing, making it appropriate for repeated LLM-generated outputs that may differ in wording despite conveying similar content [29].

Because high semantic similarity does not necessarily guarantee clinically identical messaging, computational reproducibility was interpreted together with the supplementary expert-based assessment of clinically meaningful inter-iteration inconsistency.

### 2.10. Statistical Analysis

Statistical analyses were performed using IBM SPSS Statistics version 23.0 (IBM Corp., Armonk, NY, USA), while reproducibility assessments and text similarity calculations were conducted using Python (v. 3.11.4) and RStudio (v. 2023.06.1+524). Inter-rater reliability among the three pediatric allergy–immunology specialists was assessed using Fleiss’ kappa coefficient and interpreted as follows: ≤0.20, slight agreement; 0.21–0.40, fair agreement; 0.41–0.60, moderate agreement; 0.61–0.80, substantial agreement; and 0.81–1.00, almost perfect agreement.

Normality was assessed using the Shapiro–Wilk test. Parametric data are presented as mean ± standard deviation, whereas non-parametric data are reported as median (interquartile range). Categorical variables are presented as frequencies and percentages. Comparisons between healthcare professional and patient/caregiver query groups were performed using Student’s *t* test for parametric variables and the Mann–Whitney U test for non-parametric variables. Correlations were examined using Pearson or Spearman coefficients, as appropriate. Statistical significance was set at *p* < 0.05, and Bonferroni correction was applied for primary multiple comparisons. Exploratory findings were interpreted cautiously in light of multiple testing considerations. During manuscript preparation, generative AI tools (ChatGPT-4o, OpenAI) were used in limited form for English language polishing of selected paragraphs. All AI-assisted text was reviewed, edited, and verified by the authors, who take full responsibility for the final content.

## 3. Results

### 3.1. Quality Assessment Outcomes

All responses received scores of at least 3 on both the mDISCERN and GQS instruments. For healthcare professional queries, median mDISCERN and GQS scores were 4 (IQR: 3–5) for both measures. Inter-rater agreement was almost perfect for mDISCERN (κ = 0.888) and substantial for GQS (κ = 0.799). For patient/caregiver queries, median mDISCERN and GQS scores were likewise 4 (IQR: 3–5), with substantial agreement for mDISCERN (κ = 0.659) and almost perfect agreement for GQS (κ = 0.888) (Table 1). Detailed domain analysis showed that, despite high overall quality, source attribution and uncertainty acknowledgment were relatively weaker areas across responses.

### 3.2. Readability Analysis

All readability indices indicated reading levels above recommended thresholds for patient education materials. For healthcare professional queries, mean scores were 14.29 ± 0.77 for ARI, 34.21 ± 4.01 for FRES, 12.96 ± 0.69 for FKGL, and 15.77 ± 0.74 for GFR. For patient/caregiver queries, the corresponding mean scores were 12.02 ± 0.75, 47.03 ± 4.46, 10.83 ± 0.67, and 13.69 ± 0.65, respectively (Table 2).

Both groups demonstrated low readability, with healthcare professional responses consistently reflecting college-level complexity and patient/caregiver responses ranging from high-school to early-college level; neither group met the accessibility standards recommended for general audiences. Statistically significant differences were found between the mean ARI, FRES, FKGL, and GFR scores of healthcare professional queries and patient/caregiver queries (*p* < 0.001). Expected correlations among indices were confirmed: FRES scores inversely correlated with other metrics (*p* < 0.001), while ARI, FKGL, and GFR demonstrated positive intercorrelations, validating computational consistency (Table 3).

### 3.3. Understandability and Reproducibility Outcomes

Healthcare professional queries achieved a mean PEMAT understandability score of 71.80 ± 5.75%, significantly lower than the patient/caregiver group score of 80.80 ± 4.73% (*p* = 0.001). For PEMAT actionability, healthcare professional queries had a mean score of 64.20 ± 15.43%, while patient/caregiver queries scored 69.90 ± 14.29%; this difference was not statistically significant (*p* = 0.403). Overall, PEMAT evaluation demonstrated moderate understandability and actionability across both query types (Table 4).

For reproducibility, healthcare professional queries showed 86.10 ± 3.84% similarity, while patient/caregiver queries demonstrated 87.30 ± 3.68% similarity. No significant difference was found between query types for reproducibility (*p* > 0.05). These findings indicate high semantic similarity across repeated responses in both groups (Table 4).

### 3.4. Suspected Misinformation and Safety-Related Concerns

The supplementary expert-based assessment results further showed that misinformation scores were generally low across all 20 questions; most responses received a score of 1, with a smaller number receiving scores of 2 or 3 (Table 5). Safety issues were identified in 3 of 20 questions (15%), suspected factual issues in 4 of 20 questions (20%), missing medical disclaimers in all 20 questions (100%), reflecting the absence of mandatory safety warnings across both user groups, and clinically meaningful inter-iteration inconsistency in 3 of 20 questions (15%) (Table 5).

Suspected factual issues were noted in the prognostic, precaution-related, and therapeutic domains, whereas safety issues and clinically meaningful inconsistencies were concentrated in a smaller subset of responses. Inter-rater agreement for this supplementary assessment was almost perfect for safety issues (Fleiss’ κ = 0.856) and substantial for suspected factual issues (Fleiss’ κ = 0.705). Agreement for misinformation scores was lower (Fleiss’ κ = 0.154).

## 4. Discussion

In this study, we evaluated the reliability, quality, readability, understandability, reproducibility, and safety-related concerns of ChatGPT-4o responses to frequently searched questions about pediatric IEI posed by healthcare professionals and patients/caregivers. Overall, ChatGPT-4o demonstrated strong performance across validated quality measures and high semantic reproducibility. However, readability levels remained above recommended thresholds, understandability was only moderate in some contexts, and the supplementary expert-based assessment identified missing medical disclaimers, selected suspected factual issues, and clinically meaningful inconsistencies in a subset of responses. Notably, agreement for misinformation scores was lower—reflecting the inherently subjective nature of screening for nuanced misinformation in rare diseases compared with more objective quality metrics. Collectively, these findings suggest that although ChatGPT-4o may serve as a useful supplementary information source in pediatric IEI, its outputs still require cautious interpretation and clinician oversight in clinically sensitive contexts.

ChatGPT-4o achieved favorable scores on the mDISCERN and GQS evaluations, indicating that its responses were generally well structured, relevant, and of acceptable overall informational quality across both healthcare professional and patient/caregiver queries. Similar findings have been reported in other medical domains [11,30,31,32], and our results suggest that ChatGPT-4o performs comparably in this rare-disease context. However, closer review revealed that these strong overall scores should be interpreted alongside specific domain limitations; lower-scoring areas were more commonly related to source attribution and acknowledgment of uncertainty than to general clarity or structural flow.

Moreover, these quality measures do not directly establish factual accuracy or clinical safety. The linguistic fluency of the model may mask underlying risks [33,34]; in our supplementary expert-based assessment, all responses lacked a medical disclaimer, and a subset contained suspected factual issues or safety-related concerns. References to supporting evidence or clinical guidelines were often absent, particularly in responses addressing prevention strategies and clinical management.

When readability was evaluated using the ARI, FRES, FKGL, and GFR, all ChatGPT-4o responses exceeded recommended readability thresholds, indicating that the generated content required a relatively high reading level. Although patient/caregiver-directed responses were significantly easier to read than those generated for healthcare professional queries, both groups remained above the levels generally recommended for patient education materials by the American Medical Association and the National Institutes of Health [35,36]. These findings suggest that ChatGPT-4o may still produce information that is insufficiently accessible for many end users, despite its ability to generate fluent and well-organized content. This limitation potentially creates barriers to effective self-management and informed decision-making, particularly in the context of rare and complex pediatric conditions such as IEI.

Our findings are consistent with a growing body of literature showing that limited readability is a recurring limitation of LLM-generated medical content. Recent studies in asthma, pediatric ophthalmology, childhood fever, and parent-directed allergy education have likewise shown that favorable overall performance does not necessarily eliminate accessibility or safety-related concerns [9,10,11,12,18,19]. Similar concerns have been reported in studies addressing oral immunotherapy, food protein-induced enterocolitis syndrome, chronic urticaria, pediatric asthma, cardiology, dermatology, and other patient education settings, where readability levels frequently remained above recommended standards despite otherwise favorable quality assessments [11,12,37,38,39,40,41,42,43,44]. However, traditional readability formulas may overestimate complexity in medical texts due to the inherent use of polysyllabic technical terminology. Nevertheless, the low readability scores observed in our study likely reflect not only the underlying complexity of pediatric IEI itself but also a broader tendency of LLMs to prioritize completeness and fluency rather than simplicity.

The persistent failure of LLMs to meet established readability standards in specialized medical contexts suggests a structural challenge in AI-generated communication [9,10,11,12,13,14]. This ‘readability gap’ likely stems from the inherent orientation of the training data, which is heavily weighted towards professional scientific and medical literature [34,45]. Consequently, without explicit prompt-based constraints, the model defaults to a heuristic that prioritizes informational density and technical precision over linguistic accessibility. By utilizing a zero-shot prompting strategy, our study highlights this baseline behavior and underscores the necessity for ‘safety-by-design’ features in public-facing medical AI, ensuring that information is not only accurate but also cognitively accessible to layperson [46]. Whether this reflects a fundamental model limitation or a consequence of prompt design, it reinforces the absolute need for clinician mediation in the dissemination of complex health information.

Regarding PEMAT outcomes, ChatGPT-4o demonstrated moderate levels of understandability and actionability across both query types. Patient and caregiver queries achieved significantly higher understandability scores than healthcare professional queries, whereas actionability scores did not differ significantly between groups. This pattern suggests that the model was generally more successful in presenting information in a way that could be followed by nonprofessional users than in consistently translating information into clear, practical next steps. In particular, actionability appeared to remain limited when responses emphasized explanation over concrete guidance.

These findings are broadly consistent with previous studies using PEMAT to evaluate AI-generated or patient-directed health information. Prior research in oral immunotherapy, childhood myopia, cataract surgery, and reconstructive urology has similarly reported low to moderate understandability and variable actionability across different medical contexts [11,23,39,40]. In our study, moderate patient/caregiver understandability was consistent with the existing literature. However, the lower understandability observed in responses to healthcare professional queries likely reflects a combination of inherent content complexity and the model’s audience-driven linguistic escalation. ChatGPT-4o appears to adopt a professional-congruent heuristic, defaulting to higher technical density when a query is perceived to originate from a medical expert [34]. This tendency may inadvertently limit the model’s utility for non-subspecialist clinicians, for whom overly dense jargon may hinder the rapid and efficient synthesis of clinical information. The absence of visual aids, glossaries, and explicit stepwise guidance likely further limited both understandability and actionability, suggesting that additional refinement is needed if LLM-generated responses are to support users with diverse health literacy needs. Furthermore, the moderate actionability scores reinforce the necessity of clinician guidance, as users may receive adequate background information without clear direction on the essential clinical steps required for IEI management.

Reproducibility analysis showed high semantic similarity across repeated responses in both query groups, suggesting that ChatGPT-4o generally generated stable answers when the same questions were submitted on different occasions. This degree of consistency is encouraging from the perspective of informational reliability, as users may expect comparable outputs when seeking repeated guidance on the same topic. However, semantic reproducibility should not be equated with full clinical consistency. In our supplementary expert-based assessment, clinically meaningful inter-iteration inconsistency was identified in a subset of responses, indicating that answers with generally similar wording or structure may still differ in ways that could influence interpretation or decision-making.

This distinction is particularly important in pediatric IEI, where subtle differences in prognostic framing, precautionary advice, or management-related wording may carry substantial implications for families and healthcare professionals. In such contexts, even infrequent inconsistencies may affect expectations, reassurance, or clinical judgment. Accordingly, high reproducibility metrics should be interpreted as evidence of general response stability rather than as proof of interchangeable clinical meaning across iterations.

The supplementary misinformation and safety-related assessment added an important layer to the interpretation of our findings. Although most responses received low misinformation scores overall, suspected factual issues and safety-related concerns were still identified in a subset of cases (20% and 15%, respectively), and all responses lacked a medical disclaimer. These findings are clinically relevant because even infrequent inaccuracies, omissions, or overly confident wording may have disproportionate consequences in pediatric IEI, where questions often involve prognosis, preventive measures, and treatment decisions.

In addition to factual accuracy, inconsistency in prognostic or precautionary messaging may itself have important psychosocial and clinical implications for families. Families of children with IEI often depend heavily on clear, stable, and trustworthy information to navigate chronic disease management, preventive strategies, and long-term uncertainty. In this context, variable AI-generated responses may heighten anxiety, foster false reassurance [47,48,49], contribute to unnecessary restrictions, or reinforce maladaptive coping behaviors. Moreover, when such responses are presented in an authoritative tone, they may undermine confidence in clinician counseling, even though they lack the contextual nuance and individualized judgment required for specialist medical advice.

The universal absence of a recommendation to seek professional medical advice is notable, as transparency and user guidance are widely regarded as important safeguards in health-related AI use [46,47,48,49,50]. Therefore, the generally favorable performance of ChatGPT-4o across quality measures should be interpreted with caution, particularly when responses are used by patients or caregivers outside direct clinical supervision. Consequently, future LLM evaluation frameworks should move beyond factual accuracy to formally incorporate safety messaging assessments, including the presence and quality of clinical disclaimers, communication of uncertainty, and proactive referral to specialized care.

From a clinical perspective, our findings suggest that ChatGPT-4o may serve as a useful supplementary information tool for both healthcare professionals and families seeking general information about pediatric IEI. Its relatively strong performance in overall quality assessments and high semantic reproducibility indicate potential value for initial orientation, topic familiarization, and broad educational support. However, the combination of limited readability, only moderate understandability and actionability, absence of medical disclaimers, and the presence of selected factual or safety-related concerns indicates that these responses should not be relied upon as a standalone basis for clinical decisions. Rather, AI-generated information in this setting should be considered an adjunct to clinician-guided communication, with particular caution regarding prognosis, preventive strategies, or treatment-related decisions. Furthermore, highly specialized and ethically nuanced topics—such as reproductive counseling, prenatal diagnosis, and preimplantation genetic testing—remain critical areas where current LLM outputs may lack the necessary depth or precision, necessitating comprehensive genetic counseling by human experts [51,52]. In practice, the safest integration of such tools may involve clinician-supervised use in which AI-generated explanations are reviewed, corrected as needed, and translated into simplified summaries tailored to family health literacy levels.

### Strengths and Limitations

This study has several strengths. First, it provides a multidimensional evaluation of ChatGPT-4o responses regarding pediatric IEI by examining quality, readability, understandability, reproducibility, and safety-related concerns within the same framework. Second, the use of frequently searched real-world questions from both healthcare professionals and patient/caregiver groups increased the practical relevance of the analysis. Third, evaluations were performed independently by three pediatric allergy-immunology specialists, with generally high inter-rater agreement across the main assessment tools. To our knowledge, this is also the first study to examine ChatGPT-4o performance specifically in the context of pediatric IEI, a rare-disease area in which evidence on AI-generated health information remains limited [13,14,15,16,17].

Several limitations should also be acknowledged. This study evaluated only the free, publicly accessible version of ChatGPT-4o; we did not perform head-to-head benchmarking against paid versions, against other large language models (for example, Claude, Gemini, or DeepSeek), against curated clinical decision support resources, or against established peer-reviewed patient-education materials. Such comparative evaluations are a logical next step and are being planned as a follow-up study by our group. Second, the question pool was deliberately limited to 20 items to allow each query to be evaluated in depth across three iterations (60 responses in total) and across multiple complementary metrics; this design prioritized analytic depth and inter-iteration analysis over breadth, and is consistent in scope with prior published LLM evaluations in pediatric and allergy–immunology settings [11,12,17]. Although the 20 selected questions reflected common information needs and the dataset was not powered for subgroup analyses by IEI subtype or query subtopic, reproductive counseling, prenatal diagnosis, and preimplantation genetic testing represent critically important topics that warrant dedicated future evaluation; our findings should therefore be interpreted as a focused baseline rather than as a comprehensive survey of the full IEI question space. Third, only initial responses were assessed; follow-up prompting, clarification, and iterative interaction—which may improve answer quality or accessibility—were not examined. Accordingly, our findings should be interpreted as a baseline evaluation of unoptimized public-interface use rather than the maximal achievable performance of LLMs under prompt engineering, iterative dialog, or retrieval-supported workflows. Fourth, although validated readability formulas were used, these metrics may overestimate linguistic complexity because of the unavoidable use of polysyllabic medical terminology. Fifth, the mDISCERN, GQS, and PEMAT instruments were originally developed and validated for human-authored, static consumer health information rather than for dynamic conversational AI outputs; in particular, the PEMAT was designed for patient-facing educational materials, so its application to professional-oriented AI responses represents a cross-contextual use and should be interpreted with caution. More broadly, validated instruments specifically developed for LLM-generated medical content do not yet exist; we therefore relied on the most widely applied tools in the current literature and supplemented them with structured expert assessment, while recognizing that this remains a transitional methodological compromise. Sixth, although we incorporated a supplementary expert-based assessment of misinformation and safety-related concerns, we did not conduct formal item-by-item verification of every medical statement against external reference standards. Finally, because AI systems evolve rapidly, our findings represent performance at a specific point in time and may not remain stable across future model updates.

## 5. Conclusions

ChatGPT-4o demonstrated generally favorable performance in providing information about pediatric IEI across both healthcare professional and patient/caregiver queries, particularly with respect to overall quality and semantic reproducibility. However, responses were limited by high readability demands, only moderate understandability and actionability in some contexts, absent medical disclaimers, and selected factual, safety-related, and clinically meaningful inter-iteration inconsistencies. These findings suggest that ChatGPT-4o may have value as a supplementary educational resource but should not be considered a replacement for direct clinical supervision or a standalone source of clinically sensitive guidance. Future improvements should focus on enhancing readability, transparency, source attribution, and response safety while enabling adaptive adjustment of content complexity according to user needs, so that AI-generated medical information can better support patients, caregivers, and clinicians in rare pediatric disease settings.

## Figures and Tables

**Table 1:** Distribution of mDISCERN and GQS scores by query audience, with inter-rater agreement.

Metric and Question Source	Median (IQR)	Range	Fleiss’ Kappa	Agreement Level
mDISCERN Score				
Healthcare Professionals	4 (3–5)	3–5	0.888	Almost Perfect
Patient/Caregiver	4 (3–5)	3–5	0.659	Substantial
GQS Score				
Healthcare Professionals	4 (3–5)	3–5	0.799	Substantial
Patient/Caregiver	4 (3–5)	3–5	0.888	Almost Perfect

**Table 2:** (**a**) Readability Scores for Patient/Caregiver Responses (Questions 1–10). (**b**) Readability Scores for Health Professional Responses (Questions 11–20).

(**a**)					
**Question/Response**	**Automated Readability Index (ARI)**	**Flesch Reading Ease Score (FRES)**	**Flesch-Kincaid Grade Level (FKGL)**	**Gunning Fog Readability Index (GFR)**	**Interpretation**
1. What is primary immunodeficiency and how does it affect my child?	12.8	42.5	11.5	14.2	Difficult (high school to early college)
2. Can my child with PID have a normal life expectancy?	11.9	48.3	10.8	13.6	Fairly difficult (high school level)
3. Is primary immunodeficiency hereditary or genetic?	13.4	38.7	12.1	15.0	Difficult (college level)
4. What treatments are available for my child’s PID?	12.5	44.1	11.2	14.0	Difficult (high school to early college)
5. Can my child with primary immunodeficiency be cured?	12.0	46.8	10.9	13.8	Fairly difficult (high school level)
6. How can I prevent infections in my child with PID?	11.7	49.2	10.5	13.4	Fairly difficult (high school level)
7. Are there any special precautions or lifestyle changes needed for my child?	11.3	51.0	10.2	13.1	Fairly difficult (high school level)
8. How often will my child need to see the doctor or get treatments?	12.2	45.6	11.0	13.9	Difficult (high school to early college)
9. Will my child be able to go to school and participate in normal activities?	11.5	50.3	10.3	13.2	Fairly difficult (high school level)
10. What signs or symptoms should I watch for that require urgent medical attention?	10.9	53.8	9.8	12.7	Fairly difficult (high school level)
(**b**)					
**Question/Response**	**Automated Readability Index (ARI)**	**Flesch Reading Ease Score (FRES)**	**Flesch-Kincaid Grade Level (FKGL)**	**Gunning Fog Readability Index (GFR)**	**Interpretation**
11. What are the latest diagnostic criteria and guidelines for different types of IEI?	14.6	32.4	13.2	16.1	Very difficult (college level)
12. How can we differentiate between various IEI disorders based on clinical presentation and lab tests?	15.1	29.8	13.7	16.5	Very difficult (college level)
13. What genetic tests are recommended for confirming IEI diagnosis in children?	14.9	31.0	13.5	16.3	Very difficult (college level)
14. What are the current treatment options and management strategies for specific IEI types?	14.3	33.9	13.0	15.8	Very difficult (college level)
15. How effective and safe are emerging therapies such as gene therapy or hematopoietic stem cell transplantation?	15.5	28.2	14.1	17.0	Very difficult (college graduate level)
16. What are the common complications and comorbidities associated with IEI in pediatric patients?	14.0	35.6	12.7	15.5	Difficult (college level)
17. How should vaccination schedules be adjusted for children with IEI?	13.8	36.9	12.5	15.3	Difficult (college level)
18. What are best practices for infection prevention and prophylaxis in IEI patients?	13.5	38.4	12.2	15.0	Difficult (college level)
19. How can we monitor and assess immune function and disease progression in IEI over time?	14.2	34.7	12.9	15.7	Very difficult (college level)
20. What resources and support systems are available for families and caregivers of children with IEI?	13.0	41.2	11.8	14.5	Difficult (high school to early college)

**Table 3:** Correlation Analysis Among Readability Scores of ChatGPT-4o Responses.

	ARI	FRES	GFR	FKGL
ARI	1	r = −0.999 *p* < 0.001	r = 0.998 *p* < 0.001	r = 0.999 *p* < 0.001
FRES	r = −0.999 *p* < 0.001	1	r = −0.996 *p* < 0.001	r = −0.998 *p* < 0.001
GFR	r = 0.998 *p* < 0.001	r = −0.996 *p* < 0.001	1	r = 0.999 *p* < 0.001
FKGL	r = 0.999 *p* < 0.001	r = −0.998 *p* < 0.001	r = 0.999 *p* < 0.001	1

ARI: Automated Readability Index, FRES: Flesch Reading Ease Score, FKGL: Flesch-Kincaid Grade Level, GFR: Gunning Fog Readability.

**Table 4:** Comparison of Understandability, Actionability, and Reproducibility between Audience Groups.

Measure	Healthcare Professional Queries (Mean ± SD)	Patient/ Caregiver Queries (Mean ± SD)	*p*-Value
Understandability (PEMAT score)	71.80 ± 5.75	80.80 ± 4.73	0.001
Actionability (PEMAT score)	64.20 ± 15.43	69.90 ± 14.29	0.403
Reproducibility (Similarity)	86.10 ± 3.84	87.30 ± 3.68	>0.05

**Table 5:** Suspected Misinformation and Safety Assessment.

Question ID	Domain	Misinformation Score (1–5)	Safety Issue	Suspected Factual Issue	Missing Disclaimer	Inter-Iteration Inconsistency
Q1	Patient	1 (1–1)	No	No	Yes	No
Q2	Patient	2 (2–2)	Yes	Yes (prognostic)	Yes	Yes
Q3	Patient	1 (1–1)	No	No	Yes	No
Q4	Patient	1 (1–1)	No	No	Yes	No
Q5	Patient	2 (1–2)	No	Yes (therapeutic)	Yes	No
Q6	Patient	1 (1–1)	No	No	Yes	No
Q7	Patient	2 (2–2)	Yes	Yes (precaution)	Yes	Yes
Q8	Patient	1 (1–1)	No	No	Yes	No
Q9	Patient	1 (1–1)	No	No	Yes	No
Q10	Patient	1 (1–1)	No	No	Yes	No
Q11	Professional	1 (1–1)	No	No	Yes	No
Q12	Professional	1 (1–1)	No	No	Yes	No
Q13	Professional	1 (1–1)	No	No	Yes	No
Q14	Professional	1 (1–1)	No	No	Yes	No
Q15	Professional	3 (2–3)	Yes	Yes (therapeutic)	Yes	Yes
Q16	Professional	1 (1–1)	No	No	Yes	No
Q17	Professional	1 (1–1)	No	No	Yes	No
Q18	Professional	1 (1–1)	No	No	Yes	No
Q19	Professional	1 (1–1)	No	No	Yes	No
Q20	Professional	1 (1–1)	No	No	Yes	No

Misinformation scores are presented as consensus score (range across three evaluators), where 1 = no misinformation, 2 = minimal misinformation, 3 = moderate misinformation, 4 = significant misinformation, and 5 = high misinformation. “Safety issue” indicates an item endorsed by at least 2 of 3 evaluators as posing potential to (a) delay appropriate medical evaluation, (b) promote inappropriate self-management, (c) generate false reassurance, (d) misrepresent severity/prognosis/treatment, or (e) provide age-inappropriate guidance, per the operational criteria defined in Methods. “Suspected factual issue” refers to an expert-identified potential concern categorized as prognostic, precaution-related, or therapeutic and does not represent independently guideline-verified error confirmation. “Missing disclaimer” indicates absence of advice to seek clinician guidance or equivalent safety-oriented wording. “Inter-iteration inconsistency” refers to clinically meaningful variation across three repeated responses to the same question.

## Data Availability

All data generated or analyzed during this study are included in this published article and its Supplementary Information Files. The dataset containing the statistical data has been uploaded as an Excel file in the Supplementary Materials.

## Supplementary Materials

The following supporting information can be downloaded at: https://www.mdpi.com/article/10.3390/jcm15114025/s1. Supplementary File S1: Full text of the twenty study questions and the corresponding ChatGPT-4o responses (10 patient/caregiver and 10 healthcare professional queries), together with the readability formula descriptions and calculation methods used in this study (Automated Readability Index, Flesch Reading Ease Score, Flesch–Kincaid Grade Level, Gunning Fog Readability Index).
