# Supplementary material for: Performance of ChatGPT-4o in Providing Information on Pediatric Inborn Errors of Immunity: A Cross-Sectional Evaluation
Source: J Clin Med. 2026 May 22;15(11):4025. doi: 10.3390/jcm15114025 (PMC13257705; doi:10.3390/jcm15114025)
Supplement: Supplementary file 1 [file jcm-15-04025-s001.zip › jcm-4307556-supplementary.pdf]

Supplementary Material

Top 10 Questions Patients and Caregivers Commonly Ask ChatGPT Regarding Children Diagnosed with Inborn Errors of Immunity

1. What is primary immunodeficiency and how does it affect my child?
2. Can my child with PID have a normal life expectancy?
3. Is primary immunodeficiency hereditary or genetic?
4. What treatments are available for my child's PID?
5. Can my child with primary immunodeficiency be cured?
6. How can I prevent infections in my child with PID?
7. Are there any special precautions or lifestyle changes needed for my child?
8. How often will my child need to see the doctor or get treatments?
9. Will my child be able to go to school and participate in normal activities?
10. What signs or symptoms should I watch for that require urgent medical attention?

Top 10 Questions Health Professionals Commonly Ask ChatGPT About Children Diagnosed with Inborn Errors of Immunity

1. What are the latest diagnostic criteria and guidelines for different types of IEI?
2. How can we differentiate between various IEI disorders based on clinical presentation and lab tests?
3. What genetic tests are recommended for confirming IEI diagnosis in children?
4. What are the current treatment options and management strategies for specific IEI types?
5. How effective and safe are emerging therapies such as gene therapy or hematopoietic stem cell transplantation?
6. What are the common complications and comorbidities associated with IEI in pediatric patients?
7. How should vaccination schedules be adjusted for children with IEI?
8. What are best practices for infection prevention and prophylaxis in IEI patients?
9. How can we monitor and assess immune function and disease progression in IEI over time?
10. What resources and support systems are available for families and caregivers of children with IEI?

Top 10 Questions Patients and Caregivers Commonly Ask ChatGPT Regarding Children Diagnosed with Inborn Errors of Immunity

1. What is primary immunodeficiency and how does it affect my child?

Primary immunodeficiency (PID) is a condition where a child's immune system is either missing or not working properly, which makes them more susceptible to infections. It's called "primary" because it's typically caused by a genetic defect that affects the immune system's ability to defend the body against harmful invaders like bacteria, viruses, and fungi.

Here's a breakdown of how it might affect your child:

1. Increased Susceptibility to Infections

- Children with primary immunodeficiency often get frequent, severe, or unusual infections that can be harder to treat. These infections might affect the lungs, sinuses, ears, skin, or gastrointestinal system, among others.
- Sometimes, infections that are usually mild or treatable in healthy children can lead to more serious health issues in kids with PID.

2. Delayed or Poor Response to Treatments

- The usual treatments, like antibiotics or antivirals, may not work as effectively or quickly. This means infections might last longer or recur frequently.

3. Chronic or Recurring Health Issues

- Because their immune system struggles to fight off infections, children with PID might experience chronic or recurring health problems, like sinus infections, pneumonia, or ear infections.

4. Autoimmune Conditions

- In some cases, the immune system may mistakenly attack the body's own tissues, leading to autoimmune diseases. This happens because the immune system is not functioning properly, which can result in things like joint pain, skin issues, or gastrointestinal problems.

5. Developmental Impact

- If PID leads to frequent infections, it may affect your child's growth, development, or quality of life due to long-term illness or hospitalizations.

6. Treatment Options

- Treatment can vary depending on the type and severity of the immunodeficiency. Common treatments include:
  - **Immunoglobulin therapy (IVIg or SCIg)**: This helps boost the child's immune system by providing them with antibodies that their body is unable to produce on its own.
  - **Antibiotics or antiviral treatments** to prevent or treat infections.
  - **Bone marrow or stem cell transplants** (in some severe cases) to help restore the immune system.
  - **Gene therapy** is being explored for certain types of PID.

Symptoms to Watch For:

- Frequent ear, sinus, or lung infections
- Severe infections that don't respond well to standard treatments
- Growth delays or failure to thrive
- Chronic diarrhea or gastrointestinal issues
- Family history of immune-related problems

How to Manage the Condition:

- **Monitoring**: Regular check-ups with specialists, such as immunologists, are key to managing PID.
- **Preventive Measures**: Children with PID may need extra precautions to avoid infections, such as limiting exposure to sick people, keeping up with vaccinations, or taking preventive medications.
- **Early Intervention**: The earlier the condition is diagnosed and treated, the better the chance of managing the symptoms and improving quality of life.

Does this align with what you've been noticing in your child's health? Or are you looking for more specific info about types of PID?

2. Can my child with PID have a normal life expectancy?

Yes, many children with primary immunodeficiency (PID) can live a normal life expectancy, but it depends on a few factors, including the type and severity of the condition, how early it's diagnosed, and how effectively it's managed. Here's a breakdown:

Key Factors that Influence Life Expectancy:

1. **Type of PID**
   There are many different types of PID, ranging from mild forms (which might not cause many problems with early diagnosis and proper treatment) to severe, life-threatening forms (which might require intensive treatments like stem cell transplants or gene therapy). Some PIDs are manageable with regular treatments, while others may lead to chronic health problems that can affect long-term health.
   - **Milder Forms**: Conditions like selective immunoglobulin A (IgA) deficiency may not drastically affect life expectancy if managed properly.
   - **Severe Forms**: Severe combined immunodeficiency (SCID), for example, may require a stem cell transplant for the child to survive and have a good quality of life.
2. **Early Diagnosis and Treatment**
   If PID is diagnosed early and treated appropriately (such as with immunoglobulin therapy or antibiotics to prevent infections), the child can often live a healthy, normal life. Early intervention is key to avoiding complications that can shorten life expectancy.
3. **Ongoing Medical Management**
   Children with PID need regular medical care, which could include:
   - Regular immunoglobulin infusions or other immune-boosting treatments
   - Preventive antibiotics and antiviral treatments to avoid infections
   - Care from specialists like immunologists or pediatricians experienced in managing PID
4. **Complications and Associated Conditions**
   Some children with PID may develop autoimmune disorders, organ damage, or chronic infections that can impact overall health. However, with the right treatment plan, many of these risks can be mitigated.
5. **Advancements in Treatment**
   Over the last couple of decades, treatments for PID have greatly improved. Stem cell transplants, gene therapy, and more targeted immune treatments have given children with severe forms of PID a chance at a normal or near-normal life expectancy. This is especially true for conditions like SCID, where early bone marrow or stem cell transplants can be life-saving.

Life Expectancy by PID Severity:

- **Mild or Moderate PIDs** (e.g., selective IgA deficiency, mild forms of common variable immunodeficiency) can have a near-normal life expectancy if properly managed.
- **Severe PIDs** (e.g., SCID, Wiskott-Aldrich syndrome, or chronic granulomatous disease) may require intensive treatments like bone marrow transplants or gene therapy for the child to have a healthy, full life.

Preventing and Managing Infections:

Since children with PID are at higher risk for infections, managing these risks with vaccines, infection-prevention strategies (e.g., good hygiene, avoiding exposure to sick people), and regular medical treatments is crucial. This proactive approach can greatly improve quality of life and life expectancy.

Psychological and Social Aspects:

While PID can pose medical challenges, many children go on to have fulfilling lives with the right support system. Psychosocial support for the family and the child can also play a huge role in ensuring that they lead as normal a life as possible. Kids with PID might need some extra care in school, but many are able to attend regular schools, participate in extracurricular activities, and live active lives.

So, in short:

- **Yes**, many children with PID, especially if diagnosed early and treated effectively, can have a normal or near-normal life expectancy. With advances in treatments and management strategies, kids with PID are living longer and healthier lives than ever before.

Are you currently navigating this diagnosis for your child? Let me know if you'd like more details about treatments or managing specific aspects of the condition!

3. Is primary immunodeficiency hereditary or genetic?

Yes, **primary immunodeficiency (PID)** is typically **hereditary** and **genetic**. Most cases are caused by mutations in specific genes that affect the immune system's ability to function properly. These genetic defects can be inherited in different ways, depending on the type of PID. Here's a closer look at how it works:

Genetic Inheritance Patterns:

1. **X-Linked Inheritance**
   Some forms of PID are inherited in an **X-linked** pattern, meaning the gene mutation is located on the X chromosome. Since males have one X and one Y chromosome, they are more likely to be affected by these disorders. Females, who have two X chromosomes, are usually carriers, and may not show symptoms, but can pass the condition to their children.
   - Example: **X-linked agammaglobulinemia (XLA)** is a type of PID where males are typically affected, while females are carriers.
2. **Autosomal Recessive Inheritance**
   Many forms of PID are inherited in an **autosomal recessive** pattern, which means that a child must inherit two copies of the defective gene (one from each parent) to be affected. If a child inherits only one defective gene, they are a **carrier** and usually won't show symptoms, but they can pass the gene on to their children.
   - Example: **Severe combined immunodeficiency (SCID)** is often inherited in this way.
3. **Autosomal Dominant Inheritance**
   Some forms of PID follow an **autosomal dominant** inheritance pattern. In this case, a child needs to inherit just one copy of the defective gene from either parent to develop the condition.
   - Example: Some forms of **hyper IgE syndrome** or **diGeorge syndrome** may follow this pattern.
4. **De Novo Mutations**
   In some cases, PID can occur due to a **new genetic mutation** (called a "de novo" mutation) that is not inherited from either parent but arises spontaneously in the affected child's DNA. This happens without a family history of the disorder, though the mutation can still be passed down to future generations.
   - Example: **Hyper IgM syndrome** can sometimes be caused by such mutations.

Family History and Genetic Testing:

- **Family History**: If you have a family history of immunodeficiencies or other genetic disorders, there's an increased risk of passing on or inheriting PID.
- **Genetic Testing**: If there is suspicion that your child may have PID, genetic testing can confirm the specific type of immunodeficiency and reveal how it was inherited. This can be very helpful in understanding the condition and managing it long term.

Carrier Status and Genetic Counseling:

- If you're concerned about the genetic risks, especially if PID runs in the family, **genetic counseling** can help. A genetic counselor can provide information about the likelihood of passing the condition on to future children and help guide decisions about testing.

In summary, **yes, PID is usually genetic**, and its inheritance pattern can be **X-linked**, **autosomal recessive**, or **autosomal dominant**, depending on the type of PID. Genetic testing and family history play a crucial role in understanding how PID may affect your child.

Are you considering genetic testing for your child, or are you wondering about something more specific? Let me know if you need more information!

4. What treatments are available for my child's PID?

The treatment options for **primary immunodeficiency (PID)** depend on the specific type of PID your child has, the severity of their symptoms, and any associated complications. Generally, the goal of treatment is to **boost the immune system**, **prevent infections**, and **manage any existing infections or other health issues** that arise due to the weakened immune system.

Here's an overview of common treatment strategies for PID:

1. Immunoglobulin Replacement Therapy (IVIg or SCIg)

- **What it is**: Immunoglobulin (Ig) therapy is one of the main treatments for PID. Immunoglobulins are antibodies that help the immune system fight infections.
- **How it works**: In **intravenous immunoglobulin (IVIg)** therapy, your child receives a mixture of antibodies from donated blood, usually through an IV. This boosts their immune system's ability to fight off infections.
- **Subcutaneous Immunoglobulin (SCIg)**: This is an alternative to IVIg, where the immunoglobulin is administered under the skin (subcutaneously) in smaller doses.
- **Why it's important**: This treatment is crucial for children with conditions like **common variable immunodeficiency (CVID)**, **X-linked agammaglobulinemia (XLA)**, and others, helping to reduce the frequency of infections.

2. Antibiotics or Antivirals

- **What it is**: Since children with PID are prone to infections, **antibiotic therapy** is often used to prevent or treat bacterial infections. In some cases, **antiviral** or **antifungal** medications may be necessary if viral or fungal infections are a concern.
- **How it works**:
  - **Prophylactic (preventive) antibiotics** are often given to prevent infections, especially in severe forms of PID.
  - **Regular courses of antibiotics** may be used to treat recurrent or chronic infections.
- **Why it's important**: These treatments help reduce the risk of infection-related complications, which can be more severe in children with PID.

3. Stem Cell or Bone Marrow Transplantation

- **What it is**: For certain severe forms of PID, especially **Severe Combined Immunodeficiency (SCID)** or other life-threatening immunodeficiencies, a **bone marrow or stem cell transplant** may be necessary to "rebuild" the child's immune system.
- **How it works**: The child receives stem cells from a donor (usually a family member or matched unrelated donor). The new stem cells help produce healthy immune cells, potentially correcting the immunodeficiency.
- **Why it's important**: This is sometimes a **life-saving** treatment for severe PID cases where other treatments have not been effective.

4. Gene Therapy (Emerging Treatment)

- **What it is**: Gene therapy is an experimental treatment that involves altering the patient's DNA to correct the genetic defect that causes PID. It's being explored for conditions like **SCID** and other genetic immune disorders.
- **How it works**: Healthy copies of the gene responsible for the immune deficiency are introduced into the child's cells, potentially fixing the root cause of the disorder.
- **Why it's important**: This could offer a more permanent solution for certain types of PID, although it's still an emerging treatment and may not be widely available yet.

5. Stem Cell Mobilization and Gene Editing (for SCID and Severe PIDs)

- **What it is**: This is a cutting-edge treatment approach in which **gene editing techniques** like CRISPR are used to repair the genetic mutations in immune cells. This might be combined with **stem cell mobilization** to encourage the body to produce healthy immune cells.
- **Why it's important**: This may offer hope for treating severe genetic forms of PID and could be a viable alternative to traditional bone marrow transplants.

6. Growth Factors and Immunostimulants

- **What it is**: In some cases, growth factors (like **granulocyte-colony stimulating factor, or G-CSF**) may be used to stimulate the production of white blood cells, which can help fight infections.
- **How it works**: These medications can help boost the immune response in children who have issues with white blood cell production.
- **Why it's important**: These treatments can support the immune system, especially if there's a deficiency in certain immune cells.

7. Vaccinations (Careful and Targeted)

- **What it is**: Vaccines help prevent certain infections, but children with PID need to be careful about which vaccines they receive, as some live vaccines can be too risky.
- **How it works**: In some cases, **inactivated vaccines** (like the flu shot, for example) are safe and necessary. However, **live vaccines** (such as MMR or rotavirus) may be avoided in certain cases due to the risk of causing illness.
- **Why it's important**: Vaccines are essential to help protect the child from common infections, especially those that are preventable by immunization.

8. Cytokine Therapy (in specific cases)

- **What it is**: **Cytokines** are proteins that regulate immune cell activity. In some PID cases, therapies targeting certain cytokines might be used to help stimulate or modulate the immune system.
- **How it works**: Medications like **interferons** (which can boost immune cell function) or **interleukins** (which can help with immune cell signaling) may be used depending on the PID type.
- **Why it's important**: These therapies can help fine-tune the immune system's activity, especially in conditions where there's an imbalance in immune cell function.

9. Management of Complications (Autoimmune Issues or Chronic Infections)

- **What it is**: Some children with PID develop complications like **autoimmune disorders** (where the immune system attacks the body's own tissues) or **chronic infections**. These conditions need to be managed with additional treatments.
- **How it works**: Depending on the complication, treatments might include immunosuppressive drugs for autoimmune conditions or long-term management of chronic infections.
- **Why it's important**: Managing these complications effectively can help improve your child's overall health and quality of life.

10. Regular Monitoring and Supportive Care

- **What it is**: Regular visits to immunologists or pediatric specialists are key to monitoring your child's health, tracking infections, and adjusting treatments as needed. Children with PID also benefit from **psychological support** to cope with chronic illness.
- **How it works**: Ongoing follow-ups with your child's doctor will help to assess the effectiveness of treatment, screen for complications, and address any new issues that arise.
- **Why it's important**: Preventive care and close monitoring help to avoid severe complications and improve quality of life.

Conclusion: Individualized Treatment is Key

Each child's treatment plan will be unique based on the specific **type of PID** they have, the severity of their condition, and their overall health. Early diagnosis and a tailored treatment plan can significantly improve the child's outlook and help them lead a relatively normal life. Regular medical supervision and proactive management are key to minimizing infections and complications.

Is there a specific treatment option you'd like to know more about, or are you currently navigating a particular treatment plan for your child? I'd be happy to help clarify any questions or provide more info based on your situation!

5. Can my child with primary immunodeficiency be cured?

The possibility of **curing** primary immunodeficiency (PID) depends largely on the **specific type** of PID your child has. Some forms of PID can be managed effectively with treatments, but others may require more intensive interventions with a potential for a **cure**.

Here's a breakdown of the current options and possibilities:

1. Bone Marrow or Stem Cell Transplantation

- **Cure Potential**: For certain severe forms of PID, especially **Severe Combined Immunodeficiency (SCID)**, **Wiskott-Aldrich syndrome**, or **chronic granulomatous disease (CGD)**, a **bone marrow or stem cell transplant** can potentially **cure** the condition by providing a healthy immune system from a donor.
- **How it works**: The transplant involves replacing the child's defective immune cells with healthy ones from a donor. If successful, this can result in a fully functioning immune system, effectively curing the immunodeficiency.
- **Success Rate**: The success rate for bone marrow transplants has improved significantly, but the procedure comes with risks, such as rejection or graft-versus-host disease (where the donor cells attack the child's body).
- **Not Always a Cure**: Even with a transplant, some children may need ongoing treatment or have complications, but for many, it leads to a **normal, healthy life**.

2. Gene Therapy (Emerging Treatment)

- **Cure Potential**: Gene therapy is an **exciting emerging treatment** that aims to directly **fix the genetic mutation** causing the PID. This approach is still being developed and is not widely available, but for some types of PID (especially SCID), gene therapy has shown promise.
- **How it works**: In gene therapy, the child's own cells are modified to correct the defective gene responsible for the immunodeficiency. These cells are then reinfused into the body.
- **Current Status**: Gene therapy has already been successfully used for certain types of SCID, and it's showing promising results in **curing** some types of PID without the need for a transplant.
- **Still Experimental**: Gene therapy is still in the **experimental stage** for many PID types, and more research is needed to determine its long-term effectiveness and safety.

3. Immunoglobulin Replacement Therapy (IVIg or SCIg)

- **Not a Cure**: **Immunoglobulin therapy** is one of the most effective treatments for many forms of PID (like **Common Variable Immunodeficiency (CVID)**), but it's considered a **long-term management** approach, not a cure.
- **How it works**: Regular infusions of immunoglobulin (antibodies) help prevent infections, but they don't **cure** the underlying immune deficiency. The child will likely need this treatment for life to stay protected from infections.
- **Why it's important**: While it's not a cure, **IVIg or SCIg therapy** can significantly improve the quality of life and **reduce infection risk**, allowing children with PID to live normal, healthy lives.

4. Management and Symptom Control

- **No Cure, But Manageable**: For some forms of PID, like **IgA deficiency** or **milder forms of CVID**, there isn't a cure, but the disease can be **managed well** with treatments to prevent infections and minimize symptoms.
- **How it works**: This may involve **prophylactic antibiotics**, **regular monitoring**, and **immune-modulating therapies**, but the underlying genetic issue remains.

5. Other Treatments for Specific PIDs

- **Cytokine Therapy**: Some immune deficiencies may benefit from **cytokine therapy** (e.g., **interferon**), which stimulates the immune system to function better, but this is not a cure.
- **Bone Marrow Boosters**: In rare cases, **growth factors** might be used to encourage the bone marrow to produce more healthy immune cells. This can help in certain PIDs, but again, it does not fix the root cause.

Conclusion:

- **Potential Cure for Some Types of PID**: Certain severe forms of PID (like SCID or Wiskott-Aldrich syndrome) can potentially be cured with **bone marrow or stem cell transplants** or **gene therapy**.
- **Long-term Management for Others**: Many other forms of PID (like **CVID**, **IgA deficiency**) are **not curable**, but they can be **managed effectively** with treatments like **immunoglobulin therapy**, **antibiotics**, and **preventive care**. With these treatments, children with PID can often live full, active lives, though they may need ongoing care.

If your child's type of PID is one of the more severe forms, a transplant or gene therapy might offer hope for a **cure**, but if it's a milder form, treatment will likely focus on **managing infections and boosting immunity** to improve quality of life.

Would you like to know more about the specific options for your child's type of PID, or perhaps about the potential outcomes for a particular treatment? Let me know if you need further details!

6. How can I prevent infections in my child with PID?

Preventing infections is a critical part of managing **primary immunodeficiency (PID)**. Since children with PID have a weakened immune system, they are more susceptible to infections that a healthy immune system would normally fight off. The goal is to reduce exposure to infectious agents and strengthen your child's immune defenses as much as possible.

Here are several strategies to help **prevent infections** in children with PID:

1. Regular Immunoglobulin Therapy (IVIg or SCIg)

- **What it is**: Immunoglobulin therapy is one of the most important treatments for preventing infections. By providing your child with **antibodies (immunoglobulins)** from donated blood, these treatments help to fill the gap left by their immune system's inability to produce sufficient antibodies.
- **Why it helps**: Regular immunoglobulin infusions (either intravenously or subcutaneously) help prevent **recurrent infections**, such as respiratory or sinus infections, which are common in children with PID.
- **Tip**: Make sure to keep up with the prescribed treatment schedule and talk to your doctor about how to optimize the therapy.

2. Prophylactic Antibiotics

- **What it is**: In some cases, children with PID may be prescribed **prophylactic (preventive) antibiotics** to prevent common bacterial infections like ear infections, pneumonia, and sinus infections.
- **Why it helps**: These antibiotics help reduce the chance of infections occurring in the first place. The doctor may adjust the antibiotics based on the type of PID and the child's infection history.
- **Tip**: Never stop or change the antibiotic regimen without consulting your doctor, as this could leave your child vulnerable to infections.

3. Proper Hand Hygiene and Hygiene Practices

- **What it is**: Teaching your child and everyone in the household to follow **strict hand hygiene** practices can significantly reduce the spread of bacteria and viruses.
- **Why it helps**: Frequent hand washing with soap and water, especially before eating or after touching potentially contaminated surfaces, can prevent infections. Alcohol-based hand sanitizers can be a good alternative if soap and water aren't available.
- **Tip**: Encourage your child to wash their hands regularly, especially if they're playing outside, touching public surfaces, or interacting with others.

4. Avoiding Exposure to Sick People

- **What it is**: Children with PID should **limit contact** with people who have colds, the flu, or other infections.
- **Why it helps**: Since their immune system is compromised, they're more vulnerable to infections. It's important to keep them away from crowded places, daycare centers, or schools during peak flu or cold season, if possible.
- **Tip**: If possible, avoid visits to hospitals or places where there might be exposure to sick individuals. If family members are sick, make sure they follow good hygiene practices to avoid transmitting the infection.

5. Vaccinations (With Caution)

- **What it is**: Vaccinations are one of the most effective ways to prevent infections, but for children with PID, some **live vaccines** may be contraindicated due to their weakened immune system.
- **Why it helps**: Vaccines help prevent serious illnesses like pneumonia, influenza, or whooping cough, which are more dangerous for children with PID.
- **Tip**: Work with your child's doctor to ensure they receive **safe, inactivated vaccines** (like the flu shot, tetanus, and others) but **avoid live vaccines** (like MMR or rotavirus) unless specifically recommended. Your doctor will help tailor a vaccination schedule.

6. Avoiding Contaminated Food and Water

- **What it is**: Children with PID should avoid eating or drinking anything that could expose them to **bacterial or viral infections**, especially if they're immune-compromised.
- **Why it helps**: Contaminated food and water can lead to **gastrointestinal infections**, which can be especially dangerous for children with weakened immune systems.
- **Tip**: Make sure food is properly cooked, and avoid unpasteurized dairy or undercooked meats. Be cautious about the water supply, especially when traveling or in places with questionable sanitation.

7. Regular Medical Checkups and Monitoring

- **What it is**: Regular visits to your child's doctor are key to ensuring that infections are caught early and treated promptly.
- **Why it helps**: Close monitoring can detect infections before they become severe. Your child's doctor will also be able to **adjust medications** like antibiotics or immunoglobulin therapy based on their health and needs.
- **Tip**: Keep a **health diary** or log of any symptoms or infections so you can easily update the doctor during appointments.

8. Limiting Exposure to Crowds and Public Spaces

- **What it is**: Since children with PID have a higher risk of catching infections, it's best to limit exposure to **crowded places** where germs spread easily (e.g., shopping malls, public transportation, or events).
- **Why it helps**: Crowds increase the likelihood of coming into contact with someone who is sick, whether it's a cold, flu, or other contagious condition.
- **Tip**: If your child needs to be in public spaces, encourage them to wear a **mask** if recommended by their healthcare provider. Also, carry **hand sanitizer** for quick cleaning.

9. Ensuring a Healthy Diet and Proper Nutrition

- **What it is**: A well-balanced, **nutritious diet** is essential for supporting the immune system, even if the immune system is compromised.
- **Why it helps**: Good nutrition helps the body maintain strength and fight off infections. While it may not cure PID, it can enhance overall health and make the immune system more resilient.
- **Tip**: Focus on a diet rich in **fruits, vegetables, whole grains, and lean proteins**. Consider adding vitamins or supplements as recommended by your child's doctor to support immune function.

10. Promoting Physical Activity (as tolerated)

- **What it is**: Regular, moderate physical activity helps to keep your child's body strong and supports a healthy immune system.
- **Why it helps**: Exercise helps maintain a healthy weight, supports the cardiovascular system, and boosts overall well-being, which can make the immune system more effective.
- **Tip**: Make sure that physical activity is **age-appropriate** and **safe** based on your child's specific condition and any restrictions from their doctor.

11. Careful Monitoring for Early Signs of Infection

- **What it is**: Since your child's immune system may not respond as quickly to infections, it's important to **monitor for signs of infection** early, so treatment can begin promptly.
- **Why it helps**: Early treatment with antibiotics or antivirals can prevent a mild infection from becoming serious.
- **Tip**: Watch for signs like fever, cough, skin rashes, or unexplained fatigue. If you notice any of these symptoms, contact your doctor right away for advice.

Conclusion:

While children with PID are at a higher risk for infections, following these preventive strategies can help significantly reduce the chances of them becoming seriously ill. **Early diagnosis, regular medical care, good hygiene, and preventive treatments** like immunoglobulin therapy and antibiotics are key to keeping your child healthy.

If you're looking for more specific advice on managing infections for your child's particular type of PID or treatment plan, feel free to ask!

7. Are there any special precautions or lifestyle changes needed for my child?

Yes, managing a child with **primary immunodeficiency (PID)** often requires certain **precautions** and **lifestyle changes** to help minimize the risk of infections and ensure the best possible health outcomes. Here are some key considerations and adjustments that might be necessary to keep your child safe and comfortable:

1. Infection Prevention Measures

- **Hand Hygiene**: Encourage **frequent hand washing** with soap and water. Make sure your child knows to wash their hands before meals, after using the bathroom, and after playing outside. **Alcohol-based hand sanitizers** can also be useful, especially when soap and water aren't available.
- **Avoiding Sick People**: To reduce exposure to infections, limit contact with anyone who is sick. This includes **avoiding crowded places**, daycare, or school during flu or cold season, or if someone in your household is ill.
- **Avoid Live Vaccines**: Some children with PID cannot receive **live vaccines** because their immune systems are too weak to handle the live virus. Your child's doctor will help create a vaccine schedule that includes **inactivated** vaccines (like flu shots) while avoiding **live vaccines** (like MMR, chickenpox, or rotavirus).

2. Adjustments to Diet and Nutrition

- **Balanced Diet**: Ensure that your child has a **nutritious, well-balanced diet** that supports overall health. Nutrient-rich foods like **fruits, vegetables, lean proteins, and whole grains** are essential to maintain energy and immune system function.
- **Avoid Unpasteurized Foods**: Children with PID should avoid **unpasteurized** dairy products, undercooked meats, and raw eggs to reduce the risk of foodborne infections.
- **Supplements**: Depending on your child's specific condition, the doctor may recommend **vitamin or mineral supplements** (such as vitamin D or zinc) to help support the immune system.

3. Regular Medical Care and Monitoring

- **Frequent Checkups**: Children with PID will need regular visits to their **immunologist** or pediatrician to monitor their health, check for infections, and adjust treatments as necessary.
- **Immunoglobulin Therapy**: If your child receives **immunoglobulin replacement therapy (IVIg or SCIg)**, it's important to **keep up with their treatment schedule** to help prevent infections.
- **Prophylactic Antibiotics**: If your child is on **prophylactic antibiotics**, make sure to follow the prescribed regimen carefully to reduce the risk of bacterial infections.

4. Environmental Adjustments

- **Safe Play and Physical Activity**: While exercise is important for overall health, you may need to adjust your child's activity level based on their condition. Talk to your doctor about **age-appropriate activities** that are safe for your child. Avoiding exposure to **high-risk areas** (like public swimming pools) may be necessary.
- **Avoiding Crowded Areas**: Children with PID should avoid crowded places, especially during flu season. This can help limit the exposure to germs that are more likely to spread in such environments.
- **Sanitize the Home**: Keep your home as clean as possible. Use disinfectants to regularly clean high-touch surfaces like doorknobs, faucets, and toys. This is especially important if your child is prone to respiratory or gastrointestinal infections.

5. Protection During Travel

- **Precautions for Travel**: When traveling, especially internationally, extra care is needed. **Avoiding raw food** or unclean water in certain countries is important, as is ensuring that vaccines are up-to-date (with doctor approval for safe ones). Your child may need a **medical alert bracelet** to inform others of their condition in case of an emergency.
- **Travel with Medications**: Make sure you pack enough of your child's prescribed medications (like immunoglobulins or antibiotics) and bring a doctor's note explaining the treatments and any medical equipment needed (such as an infusion pump).

6. School and Social Life

- **Communicate with Schools**: If your child is school-aged, it's crucial to **communicate** with their school or daycare about their condition. The school can take steps to help prevent exposure to germs, such as **allowing extra sick days**, offering a **clean space for your child**, or **providing access to hand sanitizers**.
- **Special Accommodations**: You may need to request specific accommodations for your child, such as avoiding exposure to other children who are sick or taking extra time off during cold and flu season.
- **Socializing**: Social interaction is important, but **limiting the number of social gatherings** during peak illness seasons (such as winter) can help reduce the risk of infection. You may want to consider **smaller playgroups** or **outdoor activities** where your child has less exposure to germs.

7. Psychological Support and Emotional Health

- **Counseling**: Living with PID can be stressful, both for the child and the family. It's important to provide **emotional support** and potentially seek professional help, such as **counseling or therapy**, to help your child cope with the psychological aspects of chronic illness.
- **Social and Emotional Growth**: It's important to help your child feel as **normal as possible**, encouraging them to take part in activities they enjoy, even if it means making special adjustments (like avoiding high-risk settings). Building confidence and maintaining a **positive attitude** can help them thrive emotionally.

8. Emergency Plans

- **Emergency Protocol**: You should have a clear understanding of what to do if your child develops symptoms of an infection or experiences a health crisis. Have a plan in place for:
  - **When to contact the doctor**
  - **Signs of an infection that require immediate medical attention** (e.g., high fever, severe fatigue, difficulty breathing)
  - **How to manage medications or infusions during a crisis**
- **Carry Medical Information**: Ensure that your child has a **medical alert bracelet** or identification card that outlines their PID and treatment plan. This can be especially important if they are ever in an emergency situation and unable to communicate their needs.

9. Be Prepared for Unexpected Situations

- **Medical Emergencies**: While prevention is key, it's still important to be prepared in case of illness or an emergency. Be ready to **adjust treatment regimens** quickly and be vigilant in **early detection** of infections.
- **Stay Informed**: Regularly check for **new information or treatments** related to your child's PID. Medical advancements and new therapies may offer additional support over time.

Conclusion:

Living with PID does require **certain lifestyle adjustments** to reduce the risk of infections and to keep your child healthy. These adjustments include maintaining **strict hygiene practices**, ensuring **proper nutrition**, having a plan for **regular medical care**, and making **environmental changes** to minimize risk. At the same time, it's important to help your child maintain a **positive, active life** within the constraints of their condition.

If you have any specific concerns or would like help navigating a particular aspect of your child's care, I'm happy to provide more detailed advice!

8. How often will my child need to see the doctor or get treatments?

The frequency of doctor visits and treatments for a child with **primary immunodeficiency (PID)** can vary based on several factors, such as the **specific type** of PID, the severity of the condition, the treatments your child is receiving, and any complications that arise. However, there are some general guidelines and recommendations:

1. Regular Doctor Visits

- **Frequency**: For most children with PID, regular **check-ups with their immunologist** or **pediatrician** are essential. Typically, your child will need to see their doctor **every 3 to 6 months**.
- **Purpose**:
  - **Monitor immune system function**: The doctor will track how well your child's immune system is responding to treatments and if there are any signs of infection.
  - **Adjust medications and treatments**: Based on your child's condition, the doctor may adjust treatments, such as immunoglobulin therapy or prophylactic antibiotics.
  - **Screen for complications**: The doctor will also be vigilant for potential **complications** of PID, such as organ damage or respiratory issues.
  - **Growth and development check**: Children with PID may experience delays or issues related to growth and development, so the doctor will track this progress as well.

- **Specialized Care**: In addition to the immunologist, your child might need to see specialists like **pulmonologists** (for lung function), **ENT specialists** (for sinus or ear infections), or **gastroenterologists** (if there are digestive issues). This may increase the frequency of visits, especially if your child experiences complications.

2. Immunoglobulin Replacement Therapy (IVIg or SCIg)

- **Frequency**: If your child is receiving **immunoglobulin replacement therapy** (either through **IVIg – intravenous immunoglobulin** or **SCIg – subcutaneous immunoglobulin**), this will generally need to be administered **every 2 to 4 weeks**, depending on the doctor's recommendation.
    - **IVIg (Intravenous Immunoglobulin)**: This is typically given at a **clinic** or **hospital** setting, and the infusion lasts a few hours. Your child will likely have regular appointments for the infusion every 3 to 4 weeks.
    - **SCIg (Subcutaneous Immunoglobulin)**: This type of therapy can often be given **at home** (after proper training), usually every 1 to 2 weeks. However, some children still prefer clinic visits for this treatment, especially when starting therapy.
- **Adjustments**: The frequency of immunoglobulin infusions may be adjusted based on how well your child is responding to treatment. If they experience frequent infections or symptoms, the doctor may recommend more frequent infusions.

3. Prophylactic Antibiotics and Other Medications

- **Antibiotics**: If your child is on **prophylactic antibiotics** (to prevent infections), they may need to continue this treatment on a **daily or weekly basis**, depending on the doctor's guidance. For some types of PID, long-term antibiotic use is essential to keep infections at bay.
- **Other Medications**: If your child requires **additional medications** (e.g., antifungals, antivirals, or medications to boost the immune system), the doctor will monitor their use during regular visits, adjusting as necessary.

4. Monitoring for Infections and Complications

- **Immediate Response**: If your child shows signs of an infection (fever, cough, persistent ear pain, etc.), you may need to make **urgent appointments** with the doctor to start **early treatment** and prevent the infection from becoming more serious. This might happen more frequently if your child is prone to infections.
- **Blood Tests**: Depending on the type of PID and treatments, your child might need **blood tests** to monitor **immunoglobulin levels**, **white blood cell counts**, and other key markers. This might be done during routine visits or more frequently if infections are a concern.

5. Hospital Visits for Specialized Treatments or Procedures

- **Specialized Procedures**: In some cases, children with PID may need **hospital visits** for more complex procedures, like:
    - **Bone marrow or stem cell transplants** (if recommended for a cure),
    - **Surgical interventions** (e.g., if infections or organ damage require surgery),
    - **Intravenous treatments** that cannot be given at home.
- These types of treatments will involve **hospitalizations** or specialized clinics, and the frequency will depend on your child's condition.

6. Immunization Schedule

- **Vaccination Monitoring**: Your child's doctor will need to ensure that they are up to date with **safe, inactivated vaccines** (such as the flu shot, pneumococcal vaccine, etc.). However, for many children with PID, there may be restrictions on **live vaccines** (like MMR or rotavirus). So, visits to the doctor will be important for planning and updating the vaccination schedule.
- **Annual Flu Shots**: In addition to the usual immunization schedule, your child will likely need an **annual flu shot** (and possibly other vaccines like the pneumococcal vaccine). These will typically require an appointment once a year.

7. Lifestyle and Monitoring Adjustments

- **Special Care During Cold and Flu Season**: During the **cold and flu season**, you might need to have **more frequent check-ins** with your doctor to ensure your child remains protected, especially if they're exposed to a higher risk of infection.
- **Check for Allergies or Asthma**: Children with PID are at greater risk of **respiratory issues**, so if your child develops symptoms of allergies or asthma, they may need additional monitoring and care.

8. Mental and Emotional Health Support

- **Psychological Monitoring**: PID can be a **stressful** condition for both the child and family. As your child grows, regular visits to a **counselor or psychologist** may be recommended to help manage the emotional and social impacts of living with a chronic illness.
- **Support for Parents**: You may also benefit from counseling or support groups for parents of children with PID, where you can connect with others facing similar challenges.

Summary of Treatment/Doctor Visit Frequency:

- **Routine doctor visits**: Every **3-6 months**, or more frequently if complications arise.
- **Immunoglobulin replacement therapy (IVIg/SCIg)**: Every **2-4 weeks**.
- **Prophylactic antibiotics**: Daily or weekly, based on doctor's prescription.
- **Blood tests and monitoring**: Depending on your child's PID type, blood work may be needed during routine visits, or more often if there are concerns about infections.
- **Vaccination checks**: **Annually**, especially for flu shots and other inactivated vaccines.
- **Emergency visits**: As needed if infections or complications arise.

The key to keeping your child healthy is consistent **monitoring and preventive care**, so while the visits may seem frequent at times, they help reduce the risk of infections and other serious health issues.

Would you like to know more about what to expect during these visits, or how to track and manage your child's treatment plan? Let me know if I can help!

9. Will my child be able to go to school and participate in normal activities?

Yes, many children with **primary immunodeficiency (PID)** can go to school and participate in normal activities, but it will often depend on the **specific type of PID**, how well it's being managed, and the precautions you take to reduce the risk of infections. With the right care and adjustments, your child can still lead a relatively normal life. Here are some important considerations and suggestions to help you decide what activities are safe for your child:

1. Going to School

- **School Attendance**: Most children with PID can attend **school regularly** if their condition is well-managed. However, certain factors like the severity of the PID, their current treatment plan, and the **risk of infections** at school should be considered.
- **Potential Precautions**:
  - **Avoiding sick classmates**: During **cold and flu season**, it might be best to **limit exposure** to large crowds, especially if there are ongoing outbreaks of illnesses.
  - **Hand hygiene**: Encouraging **frequent handwashing** at school is crucial. Ensure that the school has enough hand sanitizers and tissues for your child.
  - **Regular check-ins**: Having a meeting with school administrators and teachers can help create an environment where your child's needs are understood. This might involve discussing ways to reduce exposure to germs and having an emergency plan in place.
  - **Sick days**: Be proactive about keeping your child home if they show signs of infection. It's better to take time off than risk developing a more serious infection.

- **IEP or 504 Plan**: If your child's PID is affecting their ability to attend school normally, you may want to discuss creating an **Individualized Education Plan (IEP)** or a **504 Plan**. These plans provide accommodations for children with chronic health conditions. Possible accommodations may include:
    - Allowing extra sick days
    - Providing a clean, isolated area for your child to rest if they're feeling ill
    - Flexible attendance requirements

2. Participating in Extracurricular Activities

- **Sports and Physical Activity**:
    - **Moderate Exercise**: While **intense physical activity** might be a concern for some children with PID (especially if they have lung or joint issues), **moderate exercise** is important for your child's overall health.
    - **Contact Sports**: If your child participates in contact sports (like football, basketball, or wrestling), you may need to consider whether the risk of injury or infection from cuts, bruises, or crowded environments is worth it. Some children with PID may be advised to avoid these activities, but **non-contact sports** (like swimming, tennis, or walking) can be safer alternatives.
    - **Tailored Exercise Plans**: Consult with your child's doctor to see what level of activity is safe for your child based on their specific condition. For example, children with lung-related PID might need to avoid high-intensity aerobic activities, while those with other types might be fine with regular exercise.
- **School Play and Social Activities**:
    - **Playdates**: Playdates are important for your child's social development. You'll need to consider whether the child or their friends are ill before arranging playdates. It's also a good idea to choose outdoor activities where there's better ventilation and less chance of exposure to respiratory infections.
    - **Music or Drama**: If your child enjoys activities like music or drama, they can generally participate in these as long as they're well-managed. Just be sure they are in environments where hygiene is practiced (for example, wiping down instruments or props).

3. Avoiding Infection Risks

- **During Flu/COVID Season**: During flu season, or in the event of ongoing illnesses like **COVID-19**, extra precautions may be needed to keep your child safe:
    - **Masking**: Depending on the situation, your child may need to wear a mask, especially in crowded environments or when they are sick.
    - **Avoiding crowded events**: Events like school assemblies, large social gatherings, or concerts may increase the risk of infection, so it might be best to limit participation during peak infection seasons.
- **Hygiene Practices at School**:
    - **Disinfecting**: Encourage your child to disinfect their desk, classroom materials, or any shared items (like computer keyboards or sports equipment) to reduce the risk of germs.
    - **School Nurse**: Keep the school nurse informed about your child's condition. They can help track any illnesses that might be circulating and ensure your child gets extra care if needed.

4. Travel and School Trips

- If your child's school organizes **trips** (e.g., field trips, class tours), it's important to assess whether the environment will be safe for your child's condition.
    - **Travel Precautions**: When traveling, especially internationally, you should avoid areas where your child could be exposed to infections that might be more common in certain regions (like **foodborne illnesses**, **airborne diseases**, or **diseases from mosquito bites**).
    - **Medical Preparedness**: Make sure that any school trip has the necessary provisions, such as access to **medications**, **emergency contacts**, and any **special medical needs** your child may have.

5. Mental Health and Emotional Well-Being

- **Social Interaction**: Participating in **social activities** like birthday parties, sleepovers, or hanging out with friends is important for your child's emotional health. While **extra precautions** might be necessary, it's important for your child to experience a sense of **normalcy** and to engage with peers.
- **Psychological Support**: Children with chronic conditions like PID can sometimes experience **anxiety** or **depression**. Encouraging open discussions about how they feel and ensuring access to **therapy or counseling** can help address any emotional challenges they might face.

6. Education at Home

- **Distance Learning**: If there are times when it's too risky for your child to attend school (such as when they're sick or during an outbreak), consider discussing **remote learning** or **home-based education** with the school. Many schools offer accommodations to children with chronic health issues to make sure they don't fall behind in their studies.

Conclusion:

Yes, your child can absolutely go to school and engage in normal activities, with **appropriate precautions** in place. It's all about managing their condition and reducing exposure to potential infections. Working closely with the school, understanding your child's specific needs, and tailoring activities accordingly will help your child enjoy a balanced, fulfilling life.

Would you like specific suggestions on how to communicate with schools or more tips for helping your child enjoy activities safely? Feel free to ask!

10. What signs or symptoms should I watch for that require urgent medical attention?

It's really important to be vigilant about signs and symptoms that could indicate your child's **primary immunodeficiency (PID)** is causing an infection or other health issues. Early intervention can help prevent complications. While each child's situation may vary depending on the **type of PID** and their specific medical history, here's a list of common signs and symptoms to watch for that would require **urgent medical attention**:

1. Signs of Infection

Children with PID are more susceptible to infections, so it's important to act quickly if any of the following symptoms occur:

- **High Fever (≥101°F / 38.3°C)**: A fever that won't come down with normal fever-reducing medications or lasts longer than a few days could be a sign of infection.
- **Persistent Cough or Difficulty Breathing**: If your child develops a **persistent cough**, wheezing, or **difficulty breathing**, it could be a sign of a respiratory infection, which is common in PID.
- **Sore Throat or Swollen Glands**: Swollen lymph nodes or a **sore throat** that doesn't improve, especially if accompanied by fever, may indicate a bacterial or viral infection.
- **Severe Earache or Ear Drainage**: Ear infections are common in children with PID. If your child complains of **severe ear pain**, or you notice **fluid drainage** from the ear, seek medical help immediately.
- **Redness, Swelling, or Pus**: Any signs of **redness**, **swelling**, or **pus** around a wound, incision site, or infection, especially if accompanied by fever, require immediate attention.
- **Unexplained Pain**: **Severe abdominal pain**, joint pain, or **back pain** that doesn't go away could be indicative of a serious infection or a complication like an abscess.

2. Persistent or Unusual Symptoms

- **Prolonged or Recurring Infections**: If your child has **persistent infections** (e.g., sinus infections, respiratory infections, skin infections) that seem to last longer than normal or keep returning, this could be a sign that their immune system is not able to fight off the infection effectively.

- **Chronic Diarrhea or Vomiting**: If your child experiences prolonged **diarrhea** or **vomiting**, especially if they're also showing signs of dehydration (dry mouth, dizziness, low urine output), it may indicate an underlying infection or GI complication.
- **Weight Loss or Poor Appetite**: Sudden **weight loss** or a lack of interest in food, especially if it continues for more than a few days, could indicate an ongoing infection or that the immune system is struggling.
- **Unexplained Fatigue or Weakness**: Extreme fatigue that doesn't improve with rest may indicate an infection or other complication. If your child is **unable to keep up with normal activities** (such as walking or playing), it's worth checking with their doctor.

3. Neurological Symptoms

- **Severe Headache**: Persistent, severe headaches, especially when combined with fever or other symptoms, could be a sign of a **serious infection** (e.g., meningitis or encephalitis).
- **Seizures**: If your child has a **seizure**, either for the first time or a recurring one, it is a **medical emergency**. Seek immediate care.
- **Sudden Confusion or Difficulty Speaking**: If your child suddenly becomes **confused**, **disoriented**, or has difficulty **speaking**, it could be a sign of a serious infection affecting the brain, like meningitis or encephalitis.

4. Breathing Issues or Chest Pain

- **Rapid Breathing or Shortness of Breath**: If your child is breathing rapidly or seems **out of breath** after normal activities, it could be a sign of a **respiratory infection** or **lung inflammation**.
- **Chest Pain or Pressure**: If your child complains of **chest pain** or a feeling of **tightness in the chest**, it could be related to a respiratory infection or another serious condition. Seek immediate attention.
- **Bluish Color Around Lips or Fingernails**: A **bluish tint** to the lips or fingernails (cyanosis) indicates that the body isn't getting enough oxygen, which is a medical emergency requiring immediate attention.

5. Skin Symptoms

- **Rash or Skin Lesions**: If your child develops a **rash** that is spreading quickly or is accompanied by **fever**, it could be a sign of a viral or bacterial infection. Pay special attention to any **blistering** or **open wounds**.
- **Swollen Extremities**: **Swelling** in the legs, arms, or face, especially if it's sudden or severe, could be a sign of **infection** or **fluid buildup**, which may require immediate intervention.

6. Severe Abdominal Pain

- **Sharp or Severe Abdominal Pain**: Persistent, **severe stomach pain**, especially if accompanied by fever, vomiting, or diarrhea, could indicate an abdominal infection, appendicitis, or a complication from the PID.
- **Blood in Stool or Vomit**: If your child's **vomit** or **stool** contains **blood**, or if you notice **black, tarry stools**, this may indicate an internal issue such as gastrointestinal bleeding and requires immediate medical care.

7. Swollen Lymph Nodes

- **Sudden or Painful Swelling of Lymph Nodes**: Swollen **lymph nodes** (in the neck, under the arms, or groin) that are **painful** or **firm** could be a sign of an infection or, in some cases, lymphoma or other serious conditions.

8. Difficulty Walking or Moving

- **Sudden Difficulty Moving or Walking**: If your child is suddenly unable to walk, stands with difficulty, or experiences weakness in their limbs, it could indicate a **neurological issue** or an **infection** affecting the nervous system.

9. Swelling of the Neck or Throat

- **Difficulty Swallowing or Breathing**: If your child has a **swollen neck**, **throat**, or **difficulty swallowing**, it could indicate **severe infection** (such as epiglottitis or throat abscess) and requires urgent medical attention.

10. Sepsis (Systemic Infection)

Signs of **sepsis**—a severe, life-threatening body-wide infection—include:

- **Fever and chills** that don't go away
- **Rapid heart rate** or **low blood pressure**
- **Cold, clammy skin**, or **mottled appearance**
- **Confusion or altered mental status**
- **Breathing difficulty or rapid breathing**

If you notice any of these symptoms, **seek emergency care immediately**.

When to Seek Urgent Care

You should seek **urgent medical attention** if any of the following occur:

- **Fever over 101°F (38.3°C)** that does not improve with over-the-counter medication.
- **Uncontrolled or worsening cough**, wheezing, or shortness of breath.
- **Severe pain** in the abdomen, chest, or joints.
- **Sudden neurological symptoms** like confusion, seizures, or difficulty speaking.
- **Difficulty swallowing**, or pain with swallowing.
- **Persistent, unusual fatigue** or inability to engage in normal activities.
- **Unexplained bleeding**, including blood in stool, vomit, or coughing up blood.

General Advice

- If you ever **feel unsure** about whether a symptom is serious enough for urgent care, it's always better to **call your child's doctor** or visit the emergency room to get expert advice.
- Keep an **emergency plan** in place with your child's doctors for quick access to care if needed, and make sure to **update** anyone caring for your child (teachers, family, etc.) on the emergency procedures and what to do if an urgent health issue arises.

Being **proactive and vigilant** about these signs will help keep your child safe, and ensure that they get the right care quickly if anything serious happens. If you have any other concerns or need further clarification, feel free to reach out!

Top 10 Questions Health Professionals Commonly Ask ChatGPT About Children Diagnosed with Inborn Errors of Immunity

1. What are the latest diagnostic criteria and guidelines for different types of IEI?

The diagnostic criteria and guidelines for **inborn errors of immunity (IEI)**—previously referred to as **primary immunodeficiencies (PID)**—are continuously evolving as our understanding of these conditions improves. These guidelines are essential for identifying, diagnosing, and managing IEIs in children and adults. Below are the **latest diagnostic criteria and guidelines**, based on updated knowledge, and organized by the types of IEIs.

1. General Diagnostic Approach to IEIs

The diagnosis of IEI is typically based on:

- **Clinical Evaluation**: Detailed patient history, including family history of immune system disorders, infections, autoimmune diseases, or malignancies.
- **Immunologic Evaluation**: Laboratory tests to assess immune function, such as **immunoglobulin levels**, **T-cell function**, and **B-cell numbers**.
- **Genetic Testing**: With advancements in **genomic sequencing**, whole exome sequencing (WES), and other high-throughput techniques, **genetic testing** is now a key part of the diagnostic process for many IEIs.

2. Key Diagnostic Criteria for Specific Types of IEI

A. Antibody Deficiencies (Humoral Immunodeficiencies)

These are the most common form of IEIs and typically result in recurrent infections, especially **respiratory and ear infections**, due to inadequate antibody production.

- **Common Variable Immunodeficiency (CVID)**:
  - **Diagnosis**: CVID is diagnosed when a person has:
    - **Low levels of two or more immunoglobulin classes (IgG, IgA, IgM)**.
    - **Recurrent infections**.
    - **Exclusion of other causes** for hypogammaglobulinemia.
    - **Failure to produce adequate specific antibodies** after immunization.
    - **Genetic testing**: In some cases, mutations in genes like **TNFRSF13B (TACI)** and **CD19** can confirm the diagnosis.
  - **Updated Guidelines** (2019): The **European Society for Immunodeficiencies (ESID)** and **World Health Organization (WHO)** updated diagnostic criteria to include more **genetic findings** and a broader definition of **autoimmunity and malignancy** in CVID.
- **X-linked Agammaglobulinemia (XLA)**:
  - **Diagnosis**: The hallmark is the **absence of B-cells** in the blood. Additionally:
    - **Low or undetectable immunoglobulin levels**.
    - **No or very few B-cells** (flow cytometry).
    - **Mutation** in the **BTK gene** on the X chromosome, usually confirmed by **genetic testing**.
    - **Family history**: XLA is typically inherited in an **X-linked recessive pattern**, so a family history of the disorder is significant.

B. T-cell Deficiencies (Cellular Immunodeficiencies)

T-cell deficiencies are often associated with **recurrent viral** and **fungal infections** and can lead to **autoimmunity** or **lymphoproliferative diseases**.

- **Severe Combined Immunodeficiency (SCID)**:
  - **Diagnosis**: SCID is diagnosed through:
    - **Severe reduction or absence of T-cells**, as shown by **flow cytometry**.
    - **Absence or severe reduction of the T-cell receptor excision circles (TRECs)**, which are used as a marker for T-cell production in newborn screening.
    - **Low or absent B-cell function** (due to lack of T-cell help).
    - Genetic testing: Mutations in **IL2RG**, **ADA**, **RAG1/2**, **JAK3**, and other genes may confirm the diagnosis.
    - **Newborn screening** for SCID has been implemented in many countries as part of routine screening.
- **DiGeorge Syndrome (22q11.2 Deletion Syndrome)**:
  - **Diagnosis**: The critical criteria are:
    - **Hypocalcemia**, **cardiac defects**, **thymic hypoplasia**, and **craniofacial anomalies**.
    - **Reduced or absent T-cell count** (due to thymic aplasia or hypoplasia).
    - **Chromosomal testing** (FISH or microarray) for the **22q11.2 deletion**.

C. Phagocytic Deficiencies

These conditions affect the **cells responsible for engulfing and destroying pathogens**, leading to a higher susceptibility to bacterial and fungal infections.

- **Chronic Granulomatous Disease (CGD)**:
  - **Diagnosis**: Characterized by a defect in **phagocyte NADPH oxidase**, leading to an inability to produce reactive oxygen species (ROS) to kill certain bacteria and fungi.
    - **Flow cytometry** to assess **oxidative burst** in neutrophils and other phagocytes.
    - **Genetic testing** for mutations in **CYBB** or other components of the NADPH oxidase complex.
    - **NBT test (Nitroblue Tetrazolium test)** and **DHR (dihydrorhodamine)** test can also be used in the diagnosis.

D. Complement Deficiencies

Deficiencies in the **complement system** lead to increased susceptibility to infections, especially with **Neisseria** species.

- **C3 Deficiency**:
  - **Diagnosis**: Low levels of **complement C3** in blood serum.
  - **C3 convertase function** may also be tested, along with testing for **C4**, **C1q**, and other components.
  - **Genetic testing**: Mutations in genes encoding **C3**, **C2**, and **C4**.

E. Autoimmunity and Overactive Immune Responses

Some IEIs are characterized by inappropriate or excessive immune responses, leading to **autoimmune diseases**, **inflammatory disorders**, and **systemic complications**.

- **Autoimmune Polyendocrinopathy-Candidiasis-Ectodermal Dystrophy (APECED)**:
  - **Diagnosis**: Associated with **mutations in the AIRE gene**, leading to **autoimmune** manifestations like **candidiasis** and **endocrine dysfunction**.
    - **Autoantibodies** to **anti-thyroid** or **anti-adrenal** may be present.
    - **Genetic testing** is the gold standard for confirming the diagnosis.
- **Hyper-IgE Syndrome (Job Syndrome)**:
  - **Diagnosis**: Characterized by elevated **IgE** levels, recurrent **staphylococcal infections**, and **eczema**.
    - **Genetic testing** for **STAT3 mutations**.
    - **Eosinophilia** may also be present.

3. Updated Guidelines for Diagnosis

The most recent diagnostic guidelines for IEIs focus on **genetic testing** as a central part of diagnosis. Below are updates from major professional societies:

- **European Society for Immunodeficiencies (ESID)**: ESID published **revised guidelines in 2020** that incorporate **genetic testing** and emphasize the role of **next-generation sequencing (NGS)** in diagnosing complex IEIs. The guidelines recommend:
  - **Comprehensive immunologic workup**: Immunoglobulin levels, B and T cell subsets, and T-cell function tests.
  - **Genetic analysis**: Especially using **whole exome sequencing (WES)** or **targeted gene panels** for specific disorders.
  - **Classification of IEIs**: Based on the **International Union of Immunological Societies (IUIS)** classification system, which categorizes IEIs based on their pathophysiological mechanisms.
- **American Academy of Allergy, Asthma, and Immunology (AAAAI)**: In the U.S., the latest **2020 guidelines** encourage the use of **early genetic screening** for **SCID** and other severe defects like **Wiskott-Aldrich syndrome**, **X-linked agammaglobulinemia**, and **autoinflammatory disorders**.

    - **Immunodeficiency Screening**: The guidelines support the use of **newborn screening** for **SCID** in all states.
    - **Genetic and functional testing** is recommended for **unexplained** recurrent infections or autoimmune diseases.
- **World Health Organization (WHO)**: The **WHO** now includes **genetic testing** as part of the criteria for diagnosing IEIs, specifically in the context of **global health programs** focused on the diagnosis of **inborn errors** that might be treated with **gene therapy** or **stem cell transplantation**.

Conclusion

The diagnostic criteria for IEIs have become more sophisticated and now rely heavily on **genetic testing** and **immunologic assessments** to pinpoint specific mutations and immune system defects. With advancements in **genomic sequencing**, we can now diagnose many types of IEIs earlier and more accurately. These guidelines also emphasize the importance of **early detection** and **targeted treatments** (e.g., **gene therapy**, **immunoglobulin replacement**, or **bone marrow transplantation**) for improving outcomes.

If you are concerned about a specific IEI, it's important to discuss the latest testing and diagnostic options with your healthcare provider. Would you like to explore diagnostic tests or treatment options for a specific type of IEI? Let me know!

2. How can we differentiate between various IEI disorders based on clinical presentation and lab tests?

Differentiating between **inborn errors of immunity (IEI)** disorders can be challenging due to the wide range of clinical presentations and overlapping symptoms. However, careful attention to clinical features, laboratory findings, and genetic testing can help clinicians make an accurate diagnosis.

1. Clinical Features in IEI

IEIs can manifest with a wide variety of clinical symptoms, including recurrent infections, autoimmune phenomena, lymphoproliferation, and even malignancy. The nature of these clinical manifestations can guide physicians toward the specific type of IEI.

A. Antibody Deficiencies (Humoral Immunodeficiencies)

These disorders primarily affect **B-cells** or **immunoglobulin production**, leading to increased susceptibility to infections, particularly with **extracellular bacteria** like **Streptococcus** and **Haemophilus**.

- **Common Variable Immunodeficiency (CVID)**:
    - **Clinical Presentation**:
        - Recurrent **sinus infections**, **respiratory infections** (pneumonia, bronchitis).
        - **Chronic diarrhea**, **autoimmune diseases** (e.g., **autoimmune hemolytic anemia**), and **lymphoma**.
        - **Eczema** or **dermatitis** in some cases.
    - **Lab Findings**:
        - Low **IgG**, **IgA**, and **IgM** levels.
        - **Impaired vaccine response** (no production of specific antibodies after immunization).
        - **Normal B-cell count** but impaired function.
    - **Genetic Testing**: While not always definitive, mutations in **TNFRSF13B (TACI)**, **CD19**, or **BAFF-R** may be identified in some patients.
- **X-linked Agammaglobulinemia (XLA)**:
    - **Clinical Presentation**:
        - Severe, recurrent **bacterial infections** (respiratory, skin, ear infections) starting in the first few months of life.
        - **Absence of tonsils** and **lymph nodes**.
        - **Chronic ear infections** leading to hearing loss in some children.

- **Lab Findings**:
    - **Undetectable or very low levels of immunoglobulins** (**IgG, IgA, IgM**).
    - **Absence or near absence of B-cells** in peripheral blood (flow cytometry).
- **Genetic Testing**: Mutation in the **BTK** gene (Bruton's tyrosine kinase).

B. T-cell Deficiencies (Cellular Immunodeficiencies)

Defects in **T-cells** or **thymic function** lead to increased susceptibility to **viral infections**, **fungal infections**, and **opportunistic infections**.

- **Severe Combined Immunodeficiency (SCID)**:
    - **Clinical Presentation**:
        - Severe **recurrent infections** starting in early infancy (e.g., **pneumonia**, **sepsis**, **candidiasis**).
        - **Failure to thrive** and **growth retardation**.
        - **No lymphoid tissue (e.g., absent tonsils, adenoids)** and **poor response to vaccines**.
    - **Lab Findings**:
        - **Absent T-cells** (flow cytometry), **low or absent TRECs** (T-cell receptor excision circles) on newborn screening.
        - **Absence of a normal immune response** to vaccines.
    - **Genetic Testing**: Mutations in **IL2RG**, **ADA**, **RAG1/2**, **JAK3**, or other SCID-related genes.
- **DiGeorge Syndrome (22q11.2 Deletion Syndrome)**:
    - **Clinical Presentation**:
        - **Congenital heart defects** (e.g., **Tetralogy of Fallot**, **hypoplastic left heart**).
        - **Hypocalcemia** and **neonatal seizures**.
        - **Craniofacial abnormalities** (e.g., low-set ears, cleft palate).
        - **Recurrent infections** due to thymic hypoplasia or aplasia.
    - **Lab Findings**:
        - **Low T-cell count**.
        - Normal **B-cells**, but **poor T-cell function**.
    - **Genetic Testing**: **22q11.2 deletion** by **FISH** or **array CGH**.

C. Phagocytic Deficiencies

Phagocytic defects impair the ability of **neutrophils**, **macrophages**, and other cells to clear infections, leading to recurrent bacterial and fungal infections.

- **Chronic Granulomatous Disease (CGD)**:
    - **Clinical Presentation**:
        - **Recurrent bacterial** and **fungal infections**, particularly with **catalase-positive organisms** like **Staphylococcus aureus**, **Aspergillus**, and **Serratia marcescens**.
        - Formation of **granulomas** in various organs (e.g., lungs, liver, lymph nodes).
    - **Lab Findings**:
        - **Impaired oxidative burst** in neutrophils (tested using **NBT or DHR** test).
        - **Normal white blood cell count** but ineffective phagocytosis.
    - **Genetic Testing**: Mutations in genes encoding **NADPH oxidase** components (e.g., **CYBB**, **CYBA**, **NCF1**).

D. Complement Deficiencies

Complement deficiencies result in increased susceptibility to **Neisseria** infections and may lead to immune complex diseases like **lupus**.

- **C3 Deficiency**:
    - **Clinical Presentation**:
        - **Recurrent infections** with **encapsulated bacteria** (e.g., **Streptococcus pneumoniae**, **Haemophilus influenzae**).
        - **Neisseria infections** (e.g., **meningitis**, **gonorrhea**) are particularly common.

- **Lab Findings**:
  - **Low C3 levels** in serum.
  - **Normal or low C4 levels**.
  - **Increased susceptibility to bacterial infections**.
- **Genetic Testing**: Mutations in the **C3 gene**.

E. Autoimmunity and Inflammatory Disorders

Some IEIs manifest with autoimmune diseases, chronic inflammation, and other systemic complications.

- **Autoimmune Polyendocrinopathy-Candidiasis-Ectodermal Dystrophy (APECED)**:
  - **Clinical Presentation**:
    - **Chronic mucocutaneous candidiasis**.
    - **Hypoparathyroidism** and **adrenal insufficiency** (Addison's disease).
    - **Ectodermal changes**, such as **nail dystrophy** and **alopecia**.
  - **Lab Findings**:
    - **Elevated autoimmune antibodies**, including **anti-thyroid** or **anti-adrenal** antibodies.
  - **Genetic Testing**: Mutations in the **AIRE gene**.
- **Hyper-IgE Syndrome (Job Syndrome)**:
  - **Clinical Presentation**:
    - **Eczema**, **elevated IgE levels**, and **recurrent staphylococcal infections**.
    - **Cold abscesses** and **osteopenia**.
  - **Lab Findings**:
    - **High IgE levels**.
    - **Eosinophilia** and recurrent **skin infections** (especially staph).
  - **Genetic Testing**: **STAT3 mutations**.

---

2. Differentiating IEIs Based on Lab Tests

Below are key lab tests that can help differentiate between various IEIs:

- **Flow Cytometry**:
  - Used to assess **B-cell** and **T-cell** populations, and can identify specific defects in cell subsets.
  - Can diagnose disorders like **XLA**, **SCID**, and **DiGeorge Syndrome** (through T-cell count).
- **Immunoglobulin Levels**:
  - **CVID**: Low **IgG**, **IgA**, and **IgM**.
  - **XLA**: Very low **IgG**, **IgA**, and **IgM**, with **no B-cells**.
  - **Hyper-IgE Syndrome**: Markedly **elevated IgE**.
- **TREC (T-cell receptor excision circle) Assay**:
  - A screening tool for **SCID**, measuring the production of **TRECs**, which are decreased or absent in infants with **SCID**.
- **Oxidative Burst Test (NBT or DHR Test)**:
  - Used to diagnose **Chronic Granulomatous Disease (CGD)**. An abnormal test indicates impaired phagocyte function.
- **Genetic Testing**:
  - **Whole Exome Sequencing (WES)** or **targeted gene panels** can identify mutations in genes responsible for different IEIs.
  - **FISH** for **22q11.2 deletion** (DiGeorge Syndrome).
  - **BTK gene mutation testing** for **XLA**.

3. Key Points in Differentiation

- **Infections**:
  - **Bacterial infections** (especially **encapsulated bacteria**) are more common in **antibody deficiencies** (e.g., **CVID**, **XLA**).

- **Viral infections** (especially with opportunistic viruses like **CMV** or **VZV**) are seen in **T-cell deficiencies** (e.g., **SCID**, **DiGeorge Syndrome**).
- **Fungal and recurrent skin infections** (e.g.,
-

- What genetic tests are recommended for confirming IEI diagnosis in children?
- What genetic tests are recommended for confirming IEI diagnosis in children?
- What genetic tests are recommended for confirming IEI diagnosis in children?

3. What genetic tests are recommended for confirming IEI diagnosis in children?

Genetic testing plays a critical role in confirming the diagnosis of **inborn errors of immunity (IEI)**, especially given the wide range of clinical presentations and overlapping features of different immunodeficiencies. Advances in **genomic medicine**, particularly **next-generation sequencing (NGS)**, have made genetic testing more accessible, precise, and pivotal in diagnosing many IEIs in children. Below are the **genetic tests** recommended for confirming IEI diagnoses in children, categorized by specific types of immune deficiencies:

---

1. Broad Genetic Testing Methods for IEIs

A. Whole Exome Sequencing (WES)

- **Description**: WES sequences all the protein-coding regions of the genome (the exome). It is especially useful when the specific cause of an IEI is unknown, as it can identify **novel mutations** and **rare genetic variants**.
- **When to Use**: WES is often recommended when **clinical symptoms** suggest an immune deficiency, but no clear diagnosis has been made after initial testing. It's especially helpful for diagnosing **complex or rare IEIs** that don't fit standard diagnostic criteria.
- **Advantages**: High sensitivity for identifying **novel mutations** that may not be detected by more targeted genetic tests. It is helpful for conditions like **severe combined immunodeficiency (SCID)**, **CVID**, and **diGeorge syndrome**.

B. Targeted Gene Panels

- **Description**: Targeted gene panels focus on specific genes that are known to be involved in IEIs. These panels often include genes associated with **antibody deficiencies**, **T-cell deficiencies**, and other immune system abnormalities.
- **When to Use**: These are recommended when there's a **strong clinical suspicion** for a specific type of IEI. Panels are more cost-effective and quicker than WES, but they focus only on known causative genes.
- **Examples**:
  - **SCID panel**: Includes **IL2RG**, **ADA**, **RAG1**, **RAG2**, and **JAK3** genes.
  - **CVID panel**: Includes **TNFRSF13B (TACI)**, **CD19**, and **ICOS** genes.
  - **XLA panel**: Includes the **BTK** gene for X-linked agammaglobulinemia.
  - **Chronic Granulomatous Disease (CGD) panel**: Includes **CYBB**, **CYBA**, and **NCF1** genes.

C. Next-Generation Sequencing (NGS) Panels

- **Description**: NGS technology allows for high-throughput sequencing and simultaneous analysis of multiple genes. **Immunodeficiency-specific NGS panels** can test for hundreds of immune-related genes, making it a comprehensive method for diagnosing IEIs.
- **When to Use**: NGS panels are used in children with **multiple recurrent infections** or a family history of immune deficiencies when the cause is unclear.
- **Example**: **Immunodeficiency Gene Panels** (often available through commercial labs) include **Wiskott-Aldrich Syndrome**, **HIGM syndrome**, and **hyper-IgE syndrome**.

---

2. Specific Genetic Tests for IEI Diagnosis

A. Antibody Deficiencies (Humoral Immunodeficiencies)

1. **X-linked Agammaglobulinemia (XLA)**
   - **Gene**: **BTK** (Bruton's Tyrosine Kinase)
   - **Test**: Genetic sequencing of **BTK** gene (X-linked).
   - **When to Use**: In male children with **severe recurrent infections**, absent or low **B-cells**, and low immunoglobulin levels (IgG, IgA, IgM).
2. **Common Variable Immunodeficiency (CVID)**
   - **Genes**: **TNFRSF13B (TACI)**, **CD19**, **BAFF-R**, **ICOS**, **NFKB1**, **STAT3**, **PIK3R1**, etc.
   - **Test**: Targeted sequencing or whole exome sequencing.
   - **When to Use**: In children with **recurrent infections** and **low immunoglobulin levels** (IgG, IgA, IgM), especially if there is **family history** or **autoimmune** symptoms.

B. T-cell Deficiencies (Cellular Immunodeficiencies)

1. **Severe Combined Immunodeficiency (SCID)**
   - **Genes**: **IL2RG**, **ADA**, **RAG1**, **RAG2**, **JAK3**, **CD3D**, **CD3E**, **ARTEMIS** (DCLRE1C), **ZAP70**.
   - **Test**: **Newborn screening** for **TRECs** (T-cell receptor excision circles) as an initial test, followed by **gene sequencing** for confirmation.
   - **When to Use**: In children with **severe, early-onset infections**, **failure to thrive**, and **absence of lymphoid tissue** (tonsils, lymph nodes). **Newborn screening** is now available for SCID in many regions.
2. **DiGeorge Syndrome (22q11.2 Deletion Syndrome)**
   - **Gene**: **22q11.2 deletion** (often involving **TBX1**).
   - **Test**: **FISH** (Fluorescence in situ hybridization) or **microarray CGH** (Comparative Genomic Hybridization).
   - **When to Use**: In children with **congenital heart defects**, **hypocalcemia**, **thymic hypoplasia**, and **facial abnormalities** (e.g., low-set ears, cleft palate). Testing is also recommended in **families with a history** of **DiGeorge syndrome**.

C. Phagocytic Deficiencies

1. **Chronic Granulomatous Disease (CGD)**
   - **Genes**: **CYBB**, **CYBA**, **NCF1**, **NCF2**, **RAC2**.
   - **Test**: Genetic sequencing or **oxidative burst tests** (NBT or DHR test) to evaluate phagocyte function.
   - **When to Use**: In children with **recurrent bacterial and fungal infections**, especially **catalase-positive organisms** (e.g., **Staphylococcus aureus**, **Aspergillus**).

D. Complement Deficiencies

1. **C3 Deficiency**
   - **Gene**: **C3**.
   - **Test**: Genetic sequencing of the **C3 gene**; **low C3 levels** and **normal C4** are key diagnostic indicators.
   - **When to Use**: In children with **recurrent infections** due to **encapsulated bacteria** (e.g., **Streptococcus pneumoniae**, **Neisseria meningitidis**) and **complement testing** showing **C3 deficiency**.

E. Autoimmunity and Inflammatory Disorders

1. **Hyper-IgE Syndrome (Job Syndrome)**
   - **Gene**: **STAT3**.
   - **Test**: Genetic sequencing of **STAT3**.

   - **When to Use**: In children with **recurrent staphylococcal infections**, **eczema**, and **elevated IgE levels**. It is associated with **cold abscesses**, **osteopenia**, and **growth retardation**.
2. **Autoimmune Polyendocrinopathy-Candidiasis-Ectodermal Dystrophy (APECED)**
   - **Gene**: **AIRE** (Autoimmune Regulator).
   - **Test**: Genetic sequencing of the **AIRE gene**.
   - **When to Use**: In children with **chronic mucocutaneous candidiasis**, **autoimmune endocrinopathies** (e.g., **hypoparathyroidism**, **Addison's disease**), and **ectodermal changes** (e.g., **nail dystrophy**, **alopecia**).

---

3. Specific Genetic Tests for Newborn Screening

Some countries and states have implemented **newborn screening** for specific immunodeficiencies, which can be very useful for early diagnosis:

- **SCID**: Newborn screening for **TREC** (T-cell receptor excision circle) levels is now standard in many areas. Low or absent TRECs suggest **SCID**, followed by **genetic testing** for definitive diagnosis.
- **Severe Antibody Deficiencies**: Screening for **XLA** and **CVID** is typically not part of routine newborn screening, but **early immunoglobulin tests** can help when there are concerns in the first few months of life.

---

4. Benefits of Genetic Testing in IEI

- **Confirming Diagnosis**: Genetic testing helps confirm the suspected IEI diagnosis, particularly in complex or atypical cases.
- **Personalized Treatment**: Identifying the exact genetic mutation can guide targeted therapies such as **bone marrow transplant**, **gene therapy**, or **enzyme replacement therapy**.
- **Family Planning**: Genetic testing can inform **carrier status** in family members, which is especially important for **X-linked** or **autosomal recessive** conditions.
- **Preventive Care**: Identifying an IEI allows for early interventions such as **prophylactic antibiotics**, **immunoglobulin replacement therapy**, or **vaccination adjustments** to reduce infection risk.

Conclusion

The choice of genetic test depends on the clinical presentation and the suspected IEI. **

4. What are the current treatment options and management strategies for specific IEI types?

The treatment options and management strategies for **inborn errors of immunity (IEI)** are often tailored to the specific **type of immunodeficiency** and the individual needs of the patient. Here’s a breakdown of current treatment options and management strategies for some of the **common IEI types**:

---

1. Antibody Deficiencies (Humoral Immunodeficiencies)

A. Common Variable Immunodeficiency (CVID)

- **Treatment Goals**: Preventing infections, improving immune function, and managing complications (autoimmunity, lymphoma).
- **Primary Treatment**:

- **Immunoglobulin Replacement Therapy (IVIG or SCIG)**: Lifelong therapy to replace missing antibodies and prevent infections. Patients typically receive regular infusions, either **intravenously (IVIG)** or **subcutaneously (SCIG)**.
- **Antibiotics**: Prophylactic antibiotics to prevent bacterial infections. These are particularly useful in younger children before IgG replacement therapy starts.
- **Vaccinations**: While CVID patients may not respond well to vaccines, ensuring they are up to date on any vaccines that they can respond to is important.

- **Other Management**:
  - **Treatment of Autoimmune Conditions**: Some children with CVID develop autoimmune diseases (e.g., **autoimmune hemolytic anemia**, **rheumatoid arthritis**), which may require immunosuppressive therapy.
  - **Surveillance for Lymphoma**: Regular monitoring for **lymphoproliferative disease** is important, as CVID patients are at increased risk for **lymphoma** and **gastric cancer**.

B. X-linked Agammaglobulinemia (XLA)

- **Treatment Goals**: Preventing infections, managing recurrent infections, and minimizing complications from infections.
- **Primary Treatment**:
  - **Immunoglobulin Replacement Therapy (IVIG or SCIG)**: Regular replacement of immunoglobulins to prevent life-threatening infections.
  - **Antibiotics**: Prophylactic antibiotics to reduce the risk of infections, especially respiratory tract infections.
  - **Vaccination**: XLA patients do not respond to vaccines that require a **T-cell dependent immune response**, so vaccination strategies are tailored to avoid live vaccines and focus on passive protection through **immunoglobulin** therapy.
- **Other Management**:
  - **Treating Infections**: Early recognition and prompt treatment of infections like **pneumonia** and **sinusitis** are crucial.
  - **Lymphoma Screening**: XLA patients may also be at higher risk of developing **lymphoproliferative disorders**, so surveillance is important.

---

2. T-cell Deficiencies (Cellular Immunodeficiencies)

A. Severe Combined Immunodeficiency (SCID)

- **Treatment Goals**: Correcting immune dysfunction, preventing infections, and promoting normal immune development.
- **Primary Treatment**:
  - **Hematopoietic Stem Cell Transplantation (HSCT)**: The definitive treatment for SCID, where hematopoietic stem cells from a matched donor are transplanted to restore immune function. Early transplant is crucial for a good outcome.
  - **Gene Therapy**: In some cases, **gene therapy** may be considered for certain forms of SCID, particularly for **X-linked SCID** (IL2RG mutations).
  - **Immunoglobulin Replacement Therapy (IVIG)**: While awaiting transplantation, immunoglobulin replacement may help prevent infections.
  - **Antibiotic Prophylaxis**: Prophylactic **antibiotics** and **antifungals** to prevent infections, especially in the first year of life.
  - **Bone Marrow Transplantation (BMT)**: For infants with SCID, early transplantation offers the best chance for survival and immune reconstitution.
- **Other Management**:
  - **Live Vaccines** should be avoided in SCID patients.
  - **Enzyme Replacement Therapy (for ADA-SCID)**: Enzyme replacement with **pegylated ADA** (adenosine deaminase) can be used in **ADA-SCID** patients to manage the underlying metabolic defect.

B. DiGeorge Syndrome (22q11.2 Deletion Syndrome)

- **Treatment Goals**: Correcting immune deficits, treating congenital heart defects, and addressing other systemic abnormalities.
- **Primary Treatment**:
  - **Thymic Transplantation**: In children with **thymic hypoplasia**, **thymic transplantation** may be considered, especially for patients with severe T-cell deficiency.
  - **Immunoglobulin Replacement Therapy (IVIG or SCIG)**: Administered if there is significant immunodeficiency.
  - **Antibiotic Prophylaxis**: Preventing infections with antibiotics, especially in the first year of life.
- **Other Management**:
  - **Cardiac Surgery**: Early **surgical intervention** for congenital heart defects (e.g., **Tetralogy of Fallot**, **hypoplastic left heart**).
  - **Calcium Supplementation**: For **hypocalcemia** (low calcium levels), **calcium supplementation** is essential.

---

3. Phagocytic Deficiencies

A. Chronic Granulomatous Disease (CGD)

- **Treatment Goals**: Preventing infections, reducing inflammation, and managing complications like granulomas.
- **Primary Treatment**:
  - **Prophylactic Antibiotics**: Daily **antibiotics** to prevent bacterial and fungal infections. Often, **trimethoprim-sulfamethoxazole** is used.
  - **Interferon-gamma Therapy**: **Interferon gamma (IFN-γ)** can be used to boost phagocyte function and reduce infection rates.
  - **Granuloma Management**: If granulomas develop (in lungs, liver, etc.), **steroids** or **surgery** may be required.
  - **Gene Therapy**: Experimental **gene therapy** for CGD is being investigated and may be a future treatment option.
- **Other Management**:
  - **Bone Marrow Transplantation (BMT)**: In some cases, a **stem cell transplant** may be considered for CGD, especially for severe or refractory cases.

---

4. Complement Deficiencies

A. C3 Deficiency

- **Treatment Goals**: Preventing infections and managing autoimmune or inflammatory manifestations.
- **Primary Treatment**:
  - **Prophylactic Antibiotics**: To prevent infections with **encapsulated bacteria** (e.g., **Streptococcus pneumoniae**, **Neisseria meningitidis**).
  - **C3 Replacement Therapy**: In some cases, **C3 replacement** therapy (e.g., **C3 concentrate**) may be used, although this is not widely available or standard.
  - **Vaccinations**: Patients should receive **vaccines** for **pneumococcus** and **meningococcus** to help reduce infection risk.
- **Other Management**:
  - **Immunosuppressive Therapy**: For autoimmune complications, immunosuppressive medications such as **steroids** or **methotrexate** may be used.

---

5. Autoimmunity and Inflammatory Disorders

A. Hyper-IgE Syndrome (Job Syndrome)

- **Treatment Goals**: Control infections, manage eczema, and prevent long-term complications like skeletal abnormalities.
- **Primary Treatment**:
  - **Prophylactic Antibiotics**: For recurrent infections, especially with **Staphylococcus aureus**.
  - **Immunoglobulin Replacement Therapy (IVIG)**: May be used if necessary to reduce infections.
  - **Corticosteroids**: For managing **eczema** and **inflammatory complications**.
  - **Antifungal Treatment**: Long-term antifungal treatment for **Candida infections** and other fungal infections.
- **Other Management**:
  - **Bone Health**: **Bisphosphonates** or **calcium/vitamin D supplementation** to prevent **osteopenia** and fractures.
  - **Steroid Sparing Agents**: **Methotrexate** or **mycophenolate mofetil** in severe inflammatory manifestations.

B. Autoimmune Polyendocrinopathy-Candidiasis-Ectodermal Dystrophy (APECED)

- **Treatment Goals**: Managing autoimmune symptoms, preventing infections, and managing endocrine dysfunction.
- **Primary Treatment**:
  - **Hormone Replacement Therapy**: For **hypoparathyroidism** (calcium and vitamin D supplementation) and **adrenal insufficiency** (corticosteroids).
  - **Antifungal Therapy**: For **chronic mucocutaneous candidiasis**, antifungal treatments (e.g., **fluconazole**) are used.
  - **Immune Modulation**: Immunosuppressive therapy if needed to control **autoimmune diseases**.
- **Other Management**:
  - **Regular Monitoring**: Ongoing monitoring for **autoimmune disorders** and **infection prevention**.

---

6. Genetic Therapies and Emerging Treatments

- **Gene Therapy**: Gene therapy is an emerging option for some IEIs like **SCID**, **CGD**, and **XLA**. It involves correcting the genetic defect by introducing a normal copy of the gene into the patient's cells.
- **CRISPR-Cas9 Technology**: In experimental stages, gene-editing technologies like **CRISPR** may offer future options for treating IEIs.

Conclusion

Management strategies for **IEIs** are highly personalized and depend on the specific defect, the age of

5. How effective and safe are emerging therapies such as gene therapy or hematopoietic stem cell transplantation?

Emerging therapies like **gene therapy** and **hematopoietic stem cell transplantation (HSCT)** are transforming the treatment landscape for **inborn errors of immunity (IEI)**. Both have shown promising outcomes, but their effectiveness and safety depend on factors such as the **type of IEI**, **age of the patient**, **timing of treatment**, and the **availability of a suitable donor** for HSCT. Here's a detailed look at these therapies:

---

1. Hematopoietic Stem Cell Transplantation (HSCT)

HSCT is a widely established treatment for certain **severe immunodeficiencies** like **severe combined immunodeficiency (SCID)**, **chronic granulomatous disease (CGD)**, and some **other IEIs**.

Effectiveness of HSCT

- **SCID**: HSCT remains the **gold standard** for treatment. The sooner the transplant is done (ideally **within the first 3–6 months of life**), the better the outcomes, especially for **T-cell reconstitution**. Children who undergo early HSCT can have a near-normal life expectancy and immune function.
  - **Success rates**: Up to **90% survival** in infants with **early diagnosis** and **immediate HSCT** for conditions like X-linked SCID, ADA-SCID, and other severe T-cell defects.
- **CGD**: HSCT can be highly effective in **correcting immune function**, with survival rates ranging from **70% to 90%**. It reduces infections and prevents long-term complications (e.g., granulomas, organ damage).
- **Other IEIs**: For some **phagocytic disorders** (e.g., **leukocyte adhesion deficiency**, **Wiskott-Aldrich syndrome**), HSCT can lead to **significant improvement** in immune function and quality of life.

Safety of HSCT

- **Risk of Rejection**: One of the primary risks of HSCT is **graft-versus-host disease (GVHD)**, where the donor immune cells attack the recipient's tissues. This is more common in **mismatched** or **haploidentical** transplants.
- **Infections**: While HSCT can cure the underlying immunodeficiency, patients are at increased risk of **infections** during the post-transplant period, particularly in the first few months, until their immune system is fully reconstituted.
- **Long-term Complications**: These include **growth delays**, **graft failure**, **secondary cancers**, and **chronic GVHD**. Ongoing surveillance is needed for potential **late effects**.

Outcomes Based on Timing

- **Early Transplantation**: The earlier the transplant is done, particularly in conditions like **SCID**, the more likely the patient is to experience **optimal immune reconstitution** and **minimal long-term complications**.
- **Late Transplantation**: If HSCT is performed after a delay (i.e., after a significant number of infections, organ damage, or developmental delay), outcomes can be less favorable, and **long-term health risks** (e.g., **autoimmunity**, **chronic infections**) may persist.

---

2. Gene Therapy

Gene therapy aims to **correct the genetic defect** causing the immunodeficiency by inserting a **functional gene** into the patient’s cells. It has shown considerable promise for several **genetic immunodeficiencies**, especially for conditions where **HSCT** may not be an option or could carry high risks.

Effectiveness of Gene Therapy

- **Severe Combined Immunodeficiency (SCID)**:
  - **X-linked SCID**: **Gene therapy** has shown excellent results, particularly in **X-linked SCID**, where the **IL2RG gene** (which encodes a critical receptor subunit for T-cells) is defective. The introduction of a functional **IL2RG gene** into **autologous stem cells** can reconstitute normal immune function.

- **Success rates**: Over **90% success** in clinical trials, with most patients developing **functional T-cells** and **B-cells**. Long-term **immune recovery** and **minimal infections** have been reported in many cases.
- **ADA-SCID**: **Gene therapy** for **ADA-SCID** (adenosine deaminase deficiency) has also shown encouraging results, particularly in **autologous stem cells** engineered to express the **ADA gene**, restoring immune function and improving quality of life.

- **Chronic Granulomatous Disease (CGD)**: Gene therapy for **CGD**, where the phagocytes have defects in generating **reactive oxygen species** to kill pathogens, has also shown great promise. By **correcting the defective gene** (e.g., **CYBB**), gene therapy restores normal phagocyte function and **reduces infections**.
  - **Success rates**: Up to **80% of patients** in clinical trials show **marked improvement** in **infection control** and **quality of life**.

Safety of Gene Therapy

- **Insertional Mutagenesis**: One of the major concerns with gene therapy is **insertional mutagenesis**, where the inserted gene accidentally disrupts normal genes, potentially leading to **leukemias** or **lymphomas**. In early trials, a small number of patients developed **T-cell leukemia** due to this problem, particularly in the context of **retroviral vector-based gene therapy**.
  - Recent advances have **minimized this risk** with **safer vector systems**, such as **lentiviral vectors**, which have a lower risk of causing insertional mutagenesis.
- **Immune Rejection**: Since gene therapy often uses the patient's own stem cells (**autologous cells**), there's typically no risk of immune rejection, which can occur in **HSCT** when using a donor's cells.
- **Long-Term Safety**: The long-term safety of gene therapy is still being studied, as many trials are in their **early stages**. However, **functional corrections** in patients with **SCID** and **CGD** are now being maintained for several years without significant side effects.

Outcomes Based on Patient Population

- **Early vs. Late Treatment**: Gene therapy is typically most effective when done early, especially for diseases like **SCID**, **CGD**, and **Wiskott-Aldrich syndrome**. If performed later in life (after **immune damage**), the benefits may be less pronounced, but some patients still show significant improvement.
- **Autologous vs. Allogeneic Stem Cells**: Most gene therapy trials currently use **autologous stem cells**, meaning the patient's own cells are used. This avoids the need for a **donor match** (a challenge in HSCT) and minimizes **immune rejection**.

---

3. Comparing HSCT vs. Gene Therapy

Aspect	HSCT	Gene Therapy
**Effectiveness**	High for early-stage IEIs (e.g., SCID)	Very high for SCID, CGD, XLA, ADA-SCID
**Safety**	Risk of GVHD, infections, late effects	Risk of insertional mutagenesis, long-term safety still being studied
**Donor Dependency**	Requires a suitable donor (HLA match)	Uses patient's own cells (autologous)
**Infection Control**	Excellent, but requires immune reconstitution	Excellent, often faster immune recovery
**Availability**	Well-established, widely available	Still in clinical trials, but increasing availability
**Cost**	High (due to donor matching, procedure, and post-care)	High (due to genetic engineering, but potentially lower long-term costs)
**Long-Term Outcome**	Good for early-stage transplants	Excellent long-term immune reconstitution for many IEIs

---

4. Future Directions and Challenges

- **Gene Editing**: With advancements in **CRISPR-Cas9** and other gene-editing technologies, it's possible to directly **edit** faulty genes within a patient's cells, potentially offering a **permanent cure** for certain IEIs without the need for external vector-based therapy.
- **Safety Improvements**: Ongoing research is focused on improving the safety profile of **gene therapy** by refining vector designs to avoid **mutagenesis** and ensuring long-term **immune tolerance** without introducing risks like cancer.
- **Expansion to Other IEIs**: While **gene therapy** has been very successful for **SCID**, **CGD**, and **XLA**, there are still many other IEIs where the application of gene therapy is in its early stages. Expanding its use to other conditions such as **CVID**, **hyper-IgE syndrome**, and **Wiskott-Aldrich syndrome** is an exciting possibility for the future.

Conclusion

Both **HSCT** and **gene therapy** are transformative treatments for specific **inborn errors of immunity (IEIs)**, with high potential for improving both **survival rates** and **quality of life**. HSCT is well-established and can offer excellent outcomes when performed early, though it comes with risks like **GVHD** and **infection** during recovery. **Gene therapy** is highly promising, especially for conditions like **SCID** and **CGD**, but it is still evolving. As both therapies continue to improve, they offer hope for patients with severe, life-threatening immune disorders.

Ongoing research into **gene therapy** and **gene editing** will

likely pave the way for even more effective and safer treatments in the near future.

6. What are the common complications and comorbidities associated with IEI in pediatric patients?

Inborn errors of immunity (IEI) in pediatric patients can lead to a range of **complications** and **comorbidities** that affect both **immune function** and **overall health**. These complications can vary greatly depending on the specific **type of IEI**, its severity, and how early it is diagnosed and treated. Below are some of the most common complications and comorbidities associated with **IEIs** in children:

---

1. Recurrent Infections

Infections are the hallmark of many IEIs and are often the first sign of an underlying immunodeficiency. These infections may be more frequent, severe, or difficult to treat compared to those in children with normal immune function.

- **Bacterial Infections**: Children with **antibody deficiencies** (e.g., **CVID**, **XLA**) are particularly prone to infections caused by **encapsulated bacteria** (e.g., **Streptococcus pneumoniae**, **Haemophilus influenzae**, **Neisseria meningitidis**).
- **Viral Infections**: Children with **T-cell deficiencies** (e.g., **SCID**, **DiGeorge syndrome**) may have an increased susceptibility to **viral infections** like **respiratory viruses** (e.g., **RSV**, **influenza**) and **cytomegalovirus (CMV)**.
- **Fungal Infections**: **Phagocytic deficiencies** (e.g., **CGD**, **leukocyte adhesion deficiency**) often lead to chronic or recurrent **fungal infections**, particularly with **Candida** and **Aspergillus species**.
- **Opportunistic Infections**: Children with **severe immunodeficiency**, such as **SCID**, are at risk for **opportunistic infections** like **Pneumocystis jirovecii pneumonia (PCP)** or **mycobacterial infections**, which can be fatal without early intervention.

---

2. Autoimmune Disorders

Several types of IEIs, particularly those involving **B-cell** or **T-cell** dysfunction, can lead to the development of **autoimmune disorders**. These may include:

- **Autoimmune Hemolytic Anemia**: Some children with **CVID** or **XLA** can develop autoimmune hemolytic anemia, where the body mistakenly attacks its own red blood cells.
- **Rheumatoid Arthritis**: **CVID** and other **immune dysregulation** disorders may predispose to the development of **rheumatoid arthritis** or **juvenile idiopathic arthritis**.
- **Systemic Lupus Erythematosus (SLE)**: Children with **CVID** or other immune dysregulation disorders may develop lupus-like symptoms, including **rash**, **joint pain**, and **renal disease**.
- **Thyroid Disorders**: Autoimmune thyroid disease, such as **Hashimoto's thyroiditis**, can occur in children with **CVID** or other IEIs.

---

3. Chronic Inflammation

Chronic inflammation is another common complication in certain IEIs, particularly in disorders where **phagocytes** or **T-cells** are defective. This can lead to the formation of **granulomas** (clusters of immune cells that form in response to infection or inflammation), tissue damage, and organ dysfunction.

- **Chronic Granulomatous Disease (CGD)**: Granulomas can form in various organs, such as the **lungs**, **liver**, and **gastrointestinal tract**, leading to chronic inflammation and damage. This can result in **chronic cough**, **abdominal pain**, and **liver enlargement**.
- **Wiskott-Aldrich Syndrome**: Children with **Wiskott-Aldrich syndrome** (WAS) may experience **inflammatory bowel disease (IBD)** and **eczema**, along with **recurrent infections**.
- **Hyper-IgE Syndrome**: **Chronic eczema** and **chronic rhinosinusitis** are common in children with **Hyper-IgE Syndrome**, along with persistent **inflammatory conditions** like **osteoporosis** or **bone fractures**.

---

4. Lymphoproliferative Disease

Lymphoproliferative diseases (i.e., abnormal proliferation of lymphocytes) can occur in certain types of IEIs, especially in **antibody deficiencies** like **CVID**. These include:

- **Lymphomas**: Children with **CVID** and **XLA** are at increased risk for developing **non-Hodgkin lymphoma** (particularly in the gastrointestinal tract) and **gastric cancer**. The immunodeficiency and chronic immune activation contribute to **lymphomagenesis**.
- **Chronic Lymphocytic Leukemia (CLL)**: Although rarer, there is an increased risk of **CLL** in **CVID** patients due to chronic immune activation and dysregulated immune surveillance.

---

5. Delayed Growth and Developmental Delays

Failure to thrive or **growth delay** can be observed in children with more severe IEIs, particularly in conditions like **SCID** or **CVID**, where chronic infections and immune dysfunction can affect overall health and growth.

- **Developmental Delays**: Some children with **severe immunodeficiencies** (e.g., **SCID**, **DiGeorge syndrome**) may experience delays in motor or cognitive development due to the **chronicity of infections**, **recurrent hospitalizations**, or **treatment side effects**.
- **Delayed Bone Growth**: **Wiskott-Aldrich syndrome** and **Hyper-IgE syndrome** can lead to **bone fragility**, **osteopenia**, and **delayed bone growth** due to chronic inflammation and **recurrent infections**.

---

6. Organ Dysfunction and Damage

Long-term or untreated immunodeficiencies can cause **irreversible organ damage**. Common examples include:

- **Pulmonary Dysfunction**: Chronic **lung infections**, **bronchiectasis**, and **respiratory failure** can develop, especially in children with **phagocytic defects** (e.g., **CGD**) or **T-cell deficiencies**. Children with **SCID** who survive past infancy are at high risk for chronic lung disease due to **recurrent respiratory infections**.
- **Renal Dysfunction**: Some children with **autoimmune disorders** associated with **CVID** or **SLE** may develop **kidney damage**, leading to **nephritis** or **renal failure**.
- **Gastrointestinal Involvement**: Disorders like **Hyper-IgE Syndrome** or **CVID** can lead to **gastrointestinal infections**, **IBD**, or **malabsorption** syndromes, leading to chronic **abdominal pain**, **diarrhea**, and **failure to thrive**.

---

7. Malignancies

As mentioned earlier, there is an increased risk of developing **malignancies**, especially in patients with **CVID**, **XLA**, or **Wiskott-Aldrich syndrome**. The risk of **lymphomas**, **gastric cancer**, and other **lymphoproliferative disorders** is higher due to chronic **immune dysregulation**.

- **Chronic immune activation** and **infections** contribute to the development of **non-Hodgkin lymphomas**, **gastric lymphoma**, and **B-cell lymphomas** in some IEIs, especially **CVID**.

---

8. Cardiovascular Issues

- **DiGeorge Syndrome** (22q11.2 Deletion Syndrome) can be associated with **congenital heart defects**, such as **tetralogy of Fallot**, **aortic arch abnormalities**, and **ventricular septal defects**, which require early **cardiac surgery** and ongoing follow-up.
- Some **phagocytic defects**, like **CGD**, can lead to chronic inflammation and scarring, which may affect **blood vessels** and lead to **vascular issues** over time.

---

9. Psychological and Social Impacts

Living with an immunodeficiency disorder can have a significant emotional and psychological impact on children and their families:

- **Chronic Illness**: Continuous hospitalizations, frequent infections, and the need for **long-term treatments** (e.g., **IVIG**, **antibiotics**) can contribute to feelings of anxiety, **social isolation**, and **depression** in both children and their families.
- **School and Social Development**: Children with IEI may face challenges in school due to frequent absences from infections or hospitalizations. They may also feel socially isolated due to being unable to engage in typical activities like sports or attending school regularly.

---

10. Bone Marrow Failure

In some IEIs, especially those with **phagocytic defects** or **Wiskott-Aldrich syndrome**, the bone marrow may fail to produce enough **blood cells** (e.g., **platelets**, **red blood cells**, **white blood cells**), leading to:

- **Anemia**: Reduced red blood cell production, leading to **fatigue**, **paleness**, and **shortness of breath**.
- **Thrombocytopenia**: Low platelet count, leading to **easy bruising**, **bleeding gums**, and **increased risk of hemorrhage**.
- **Leukopenia**: Low white blood cell count, leading to **increased susceptibility** to infections.

Conclusion

The **complications and comorbidities** associated with **inborn errors of immunity (IEIs)** are diverse and can affect virtually every organ system in the body. Early **diagnosis** and **treatment** are crucial for preventing or mitigating many of

these issues. **Immunotherapy**, **HSCT**, and **gene therapy** have greatly improved outcomes for many children with IEI, but ongoing medical care is often needed to address complications like infections, autoimmune diseases, and organ damage. **Multidisciplinary management** is often required, involving immunologists, pediatricians, infectious disease specialists, and other healthcare providers.

7. How should vaccination schedules be adjusted for children with IEI?

Vaccination is a critical aspect of managing **inborn errors of immunity (IEI)**, but it requires careful consideration and adjustments to account for the specific **immune deficiency** a child has. While vaccines are important for protecting children from preventable diseases, some **standard vaccines** may not be appropriate or effective for children with certain types of IEI. Below is a breakdown of how vaccination schedules should be adjusted for children with IEIs:

General Considerations for Vaccination in Children with IEI

1. **Assessment of Immune Function**:
   - **Immune status** should be thoroughly assessed before vaccination. For example, children with **B-cell deficiencies** (e.g., **CVID**, **XLA**) may need additional doses or different timing for some vaccines.
   - **T-cell** and **complement deficiencies** may require different vaccines or specific considerations for live vaccines.
2. **Live vs. Inactivated Vaccines**:
   - **Inactivated vaccines** (e.g., **polio (IPV)**, **Hepatitis B**, **DTaP**) are generally safe for most children with IEIs, though some children may need adjusted doses.
   - **Live vaccines** (e.g., **MMR**, **varicella**, **rotavirus**) should be avoided in certain immune-deficient children, particularly those with **severe T-cell deficiencies** (e.g., **SCID**, **DiGeorge syndrome**), due to the risk of causing disease.
3. **Timing**:
   - Some vaccines may need to be delayed until a child's immune function is stable, or after treatments like **immunoglobulin therapy** or **HSCT**. For example, **HSCT patients** should be vaccinated again after transplantation, as their immune system will be reset.
4. **Immune Globulin (IVIG) and Vaccines**:
   - **IVIG therapy** may interfere with the efficacy of **live vaccines** due to the passive antibodies that are introduced through the treatment. It's often recommended to schedule vaccines several months before or after **IVIG infusions** for optimal response.

Adjustments to Vaccination Schedule Based on Type of IEI

1. Antibody Deficiencies (e.g., CVID, XLA, Hyper-IgM Syndrome)

- **Inactivated Vaccines**: These vaccines are generally safe and effective for children with **antibody deficiencies**. However, they may require **extra doses** or **booster shots**.
    - **Examples**: **Hepatitis B**, **diphtheria**, **tetanus**, **pertussis (DTaP)**, **polio (IPV)**, **Hib**.
    - **Pneumococcal Vaccine**: Children with **CVID** should receive **pneumococcal vaccines** (e.g., **PCV13**, **PPSV23**) based on their age and condition, and sometimes may need additional **pneumococcal boosters**.
- **Live Vaccines**: **Live vaccines** (e.g., **MMR**, **varicella**, **rotavirus**) may not be appropriate for children with severe **antibody deficiencies** or those receiving **immunoglobulin therapy** because the immune system might not mount a sufficient response.
    - In these cases, live vaccines should be **delayed** until **immune function is better supported** (i.e., after **IVIG** therapy).

2. T-Cell Deficiencies (e.g., SCID, DiGeorge Syndrome)

- **Inactivated Vaccines**: These are safe and should be administered according to the typical vaccine schedule. The child may need **additional doses** or **boosters** to ensure proper protection.
    - **Examples**: **DTaP**, **polio (IPV)**, **Hepatitis B**, **Hib**, **pneumococcal**, **influenza**.
- **Live Vaccines**: **Live vaccines** (e.g., **MMR**, **varicella**, **yellow fever**) are **contraindicated** for children with severe **T-cell deficiencies** because their immune system cannot handle the live virus, which could lead to **disease** or **complications**.
    - In these cases, **live vaccines** should be **delayed** or avoided altogether until the child has adequate **immune reconstitution** (e.g., after **HSCT** or **gene therapy**).

3. Phagocytic Defects (e.g., Chronic Granulomatous Disease, Leukocyte Adhesion Deficiency)

- **Inactivated Vaccines**: Safe and should be given as per the usual schedule.
    - **Examples**: **DTaP**, **polio (IPV)**, **Hepatitis B**, **pneumococcal vaccine**, **influenza**.
- **Live Vaccines**: **Live vaccines** are generally avoided in children with **phagocytic defects**, as they are at risk of **severe infections** from live viruses, even though their immune system has some functional ability to fight bacteria.
    - **Varicella**, **MMR**, and other **live vaccines** should be **avoided**.
    - However, in **less severe cases**, consultation with an immunologist is needed to determine if **live vaccines** can be safely administered.

4. Complement Deficiencies (e.g., C3 Deficiency, Properdin Deficiency)

- **Inactivated Vaccines**: Children with **complement deficiencies** can safely receive **inactivated vaccines**, as these vaccines help stimulate the immune system without relying on **complement activation**.
    - **Examples**: **DTaP**, **polio**, **Hepatitis B**, **Hib**, **pneumococcal vaccines**, **influenza**.
- **Live Vaccines**: **Live vaccines** may be safe in some cases, but this decision should be guided by the **specific deficiency** and the child's overall immune function. For example, children with **isolated complement deficiencies** (like **C3 deficiency**) may tolerate live vaccines better than those with **severe combined T- and B-cell deficiencies**.

5. Combined Immunodeficiencies (e.g., Ataxia Telangiectasia, Wiskott-Aldrich Syndrome)

- **Inactivated Vaccines**: These vaccines should generally be given on the regular schedule. Additional doses may be needed based on the severity of the deficiency.
    - **Examples**: **DTaP**, **polio**, **Hepatitis B**, **influenza**, **pneumococcal**.
- **Live Vaccines**: **Live vaccines** may be contraindicated, especially in children with **Wiskott-Aldrich Syndrome** or **Ataxia Telangiectasia**, due to **impaired immune response**.
    - **MMR** and **varicella** vaccines should be avoided if the child is not receiving treatment that restores immune function (e.g., **HSCT**).

Vaccination Strategies and Tips for IEI Children

1. **Consult with Immunologist**: The **immunologist** should play a central role in advising on the safest vaccination schedule and providing guidance on whether certain vaccines (e.g., **live vaccines**) are safe or should be avoided.
2. **Avoid Live Vaccines if Immune Deficient**: In general, **live vaccines** should be avoided in children with **severe immunodeficiencies**, especially **T-cell deficiencies**, and should only be considered in specific cases where immune function has been restored (e.g., after **HSCT**).
3. **Use of Immunoglobulin Therapy**: For children receiving **IVIG**, the **timing** of vaccinations may need adjustment, as IVIG contains passive antibodies that can interfere with the **immune response** to **live vaccines**. Vaccines are often given **several months before or after IVIG infusions**.
4. **Boosters for Vaccine Efficacy**: Children with certain **IEIs**, particularly **antibody deficiencies**, may require **additional doses** of vaccines to achieve sufficient protection. In cases like **CVID**, **booster shots** for **pneumococcal** or **Hepatitis A** vaccines might be necessary.
5. **Vaccines for Specific IEIs**: Consider adding vaccines that target **increased risks** in specific IEIs. For example, children with **CVID** or **XLA** should be vaccinated for **pneumococcus**, **meningococcus**, and **Hib** as they are at greater risk of these infections.
6. **Document Vaccine Response**: For children with **antibody deficiencies**, it is important to **measure the immune response** after vaccination (e.g., **serologic titers**) to determine if the vaccine provided sufficient protection. If not, additional or alternative treatments (e.g., **boosters**, **antibiotics**) may be necessary.

Conclusion

Vaccination in children with **inborn errors of immunity (IEI)** requires a personalized and cautious approach. For many children, **inactivated vaccines** are safe and effective, but **live vaccines** are typically avoided in those with severe **immune deficiencies** unless the child has undergone treatments like **HSCT** that can restore immune function. Regular consultations with an **immunologist** are essential to develop an appropriate vaccine schedule and manage the risks associated with immunodeficiencies.

Early and **appropriate vaccination** can help protect these children from preventable diseases and improve their overall health and quality of life.

8. What are best practices for infection prevention and prophylaxis in IEI patients?

In **patients with inborn errors of immunity (IEI)**, the immune system's ability to fight off infections is compromised, making infection prevention and **prophylaxis** crucial parts of their care. Given that these children are more susceptible to a wide range of infections, managing these risks requires a **multidisciplinary approach** and **close monitoring**. Below are best practices for infection prevention and prophylaxis in children with IEI:

---

1. Early Diagnosis and Monitoring

- **Early Diagnosis**: Identifying IEI early, especially in the neonatal or infancy period, allows for prompt initiation of prophylactic measures before serious infections occur.
- **Regular Monitoring**: Routine monitoring of the immune system's function (e.g., **serum immunoglobulin levels**, **T-cell function**, **complete blood count**) to gauge how well the child's immune system is functioning.
- **Infection Surveillance**: Regular visits to the doctor for **screening** and to monitor **early signs of infection** (fever, cough, skin rashes, etc.) are essential.

---

2. Immunoglobulin Replacement Therapy (IVIG or SCIG)

- **Intravenous Immunoglobulin (IVIG)** or **Subcutaneous Immunoglobulin (SCIG)** therapy is commonly used in children with **antibody deficiencies** (e.g., **CVID**, **XLA**). These treatments provide broad **passive immunity** to help prevent bacterial and viral infections.
  - **Frequency**: IVIG is typically administered every **3-4 weeks**, but the frequency may vary based on the severity of the deficiency.
  - **Effectiveness**: Regular **IVIG/SCIG** infusions are essential to maintain **adequate antibody levels** and protect against infections such as **pneumonia**, **sinusitis**, **otitis media**, and **meningitis**.
  - **Prophylaxis Effect**: Studies show that IVIG reduces the frequency of infections, hospitalizations, and the need for **antibiotics** in patients with **antibody deficiencies**.

---

3. Antibiotic Prophylaxis

- **Prolonged Antibiotic Prophylaxis**: For children with **severe IEI**, especially those with **phagocytic defects** (e.g., **CGD**) or **antibody deficiencies**, antibiotics may be prescribed **long-term** to prevent infections.
  - **Common Prophylactic Antibiotics**: **Penicillin**, **amoxicillin**, **trimethoprim-sulfamethoxazole** (TMP-SMX), and **azithromycin** are commonly used to prevent infections.
  - **Pneumocystis jirovecii**: **TMP-SMX** is typically used to prevent **Pneumocystis jirovecii pneumonia (PCP)**, especially in children with **T-cell deficiencies** (e.g., **SCID**, **DiGeorge syndrome**).
  - **Fungal Prophylaxis**: In children with **phagocytic defects** like **CGD**, **antifungal agents** (e.g., **itraconazole**, **fluconazole**) may be used to prevent fungal infections.

---

4. Antiviral Prophylaxis

- **Herpes Simplex Virus (HSV)**: Children with **severe immune deficiencies** (e.g., **SCID**, **CVID**) may be at risk for **HSV** reactivation, so **antiviral agents** like **acyclovir** or **valacyclovir** can be used to prevent infections.
- **Cytomegalovirus (CMV)**: Children undergoing **hematopoietic stem cell transplantation (HSCT)** or those with **severe T-cell deficiencies** may receive **antiviral therapy** for **CMV** reactivation (e.g., **valganciclovir** or **ganciclovir**).

---

5. Vaccination Strategies

- **Inactivated Vaccines**: These are generally safe for children with IEIs, and they should be given according to an adjusted vaccine schedule. Examples include:
  - **Hepatitis B**
  - **DTaP**
  - **Polio (IPV)**
  - **Pneumococcal** (PCV13, PPSV23)
  - **Hib**
  - **Influenza**
  - **Meningococcal vaccines** (especially for **CVID** or **asplenic** patients)
- **Live Vaccines**: **Live vaccines** (e.g., **MMR**, **varicella**) should be avoided in patients with severe **T-cell deficiencies**, as they can lead to **vaccine-derived diseases**. However, in some cases, live vaccines can be safely administered after immune restoration (e.g., after **HSCT**).
- **Pneumococcal Vaccine**: This is especially important for children with **CVID**, **XLA**, and other immune deficiencies, as these children are highly susceptible to **pneumococcal infections**.

---

6. Infection Control Measures

- **Hygiene**: Practicing **good hygiene** is one of the simplest but most important ways to prevent infections in children with IEI.
  - **Handwashing**: Teach the child and family to wash hands frequently with soap and water.
  - **Avoiding Sick Contacts**: Minimize exposure to sick individuals. This is particularly important during cold and flu season.
  - **Face Masks**: In high-risk environments, **masking** (e.g., for immunocompromised children) can reduce exposure to airborne pathogens.
- **Isolation**: For children with severe IEI (e.g., **SCID** or those awaiting **HSCT**), **precautions** may be necessary to protect them from infections. This may involve:
  - **Limiting exposure** to public spaces or crowded environments.
  - **Avoiding daycare or school** during illness outbreaks (e.g., flu season, chickenpox outbreaks).
- **Environmental Measures**:
  - **Cleanliness at Home**: Ensure that home environments are **clean** and **disinfected** regularly, particularly surfaces that are often touched (e.g., doorknobs, faucets, toys).
  - **Air Quality**: In some cases, using an **air purifier** or ensuring good ventilation in the home can help reduce exposure to respiratory pathogens.

---

7. Prophylaxis for Specific Pathogens

- **Pneumococcal Infections**: Children with **CVID** and other immune deficiencies should receive **pneumococcal vaccination** and potentially **antibiotic prophylaxis** with **penicillin** or **amoxicillin**.
- **Meningococcal Infections**: **Meningococcal vaccines** (e.g., **MenACWY** and **MenB**) should be given, particularly to children with **CVID**, **asplenia**, or those undergoing **HSCT**.
- **Influenza**: Annual **influenza vaccinations** (preferably **inactivated**) are essential for preventing **influenza-related complications** in children with IEI.
- **Respiratory Infections**: For children with **T-cell deficiencies**, regular use of **antiviral prophylaxis** (e.g., **oseltamivir** for flu) or **influenza vaccinations** should be considered to reduce the risk of respiratory infections.

---

8. Nutritional Support and Supplements

- **Adequate Nutrition**: Ensure that the child is receiving a balanced diet to maintain optimal immune function. Deficiencies in essential vitamins and minerals can impair the immune response.
  - **Vitamin D**: Supplementing with **vitamin D** has been shown to enhance immune function and may reduce the risk of **respiratory infections**.
  - **Zinc**: Adequate **zinc** levels are important for immune function and wound healing.
- **Probiotics**: In some cases, **probiotics** may help maintain healthy gut flora, particularly if antibiotics are used frequently.

---

9. Close Follow-up with Healthcare Providers

- **Regular Check-ups**: **Immunology** follow-ups are crucial for monitoring treatment responses (e.g., effectiveness of **IVIG**, antibiotic prophylaxis, etc.).
- **Emergency Plan**: Have an **emergency plan** in place with clear instructions on what to do in case of an infection (e.g., fever, difficulty breathing). This plan should include contact information for the child's medical team.

---

10. Education and Support

- **Patient and Family Education**: Educate the family and caregivers on the importance of infection prevention, the need for adherence to prophylactic treatments (antibiotics, IVIG), and how to recognize early signs of infection.
- **Psychosocial Support**: Address the emotional and social impact of living with an immunodeficiency, especially for families that may be isolated due to frequent hospitalizations or infections. Providing access to counseling and peer support groups can help.

Conclusion

Infection prevention and prophylaxis are essential components of managing children with **inborn errors of immunity (IEI)**. Best practices include a combination of **immunoglobulin replacement**, **antibiotic** and **antiviral prophylaxis**, careful **vaccination schedules**, and **infection control measures** such as good hygiene and limiting exposure to sick individuals. Regular **monitoring** by an immunologist, close attention to **nutritional status**, and **psychosocial support** for the family are also crucial to ensuring the best possible outcomes for children with IEI.

9. How can we monitor and assess immune function and disease progression in IEI over time?

Monitoring and assessing immune function and disease progression in children with **inborn errors of immunity (IEI)** are vital for managing their condition and preventing infections, complications, and other health issues. Regular evaluation is key to adjusting treatment plans, ensuring that the immune system is supported appropriately, and identifying early signs of disease progression or new complications. Here's a comprehensive guide on how to monitor and assess immune function and disease progression in IEI over time:

---

1. Clinical Monitoring

Routine clinical assessments are essential to monitor how the child is responding to treatment, assess their growth and development, and detect any signs of **new or worsening infections**. These include:

- **Growth and Development**: Regular assessments to track the child's physical and developmental milestones. Growth failure can indicate **underlying infections**, poor nutrition, or other complications.
- **Symptom Monitoring**: Monitoring for any signs of infection or other complications. The child's medical team should look for symptoms like:
  - **Recurrent or persistent infections** (e.g., respiratory, gastrointestinal, or skin infections)
  - **Chronic diarrhea**, **skin rashes**, or **autoimmune symptoms** like joint pain, skin inflammation, or vasculitis.
  - **Fatigue**, **fever**, or signs of **organ involvement** (e.g., liver, kidney, or lung issues).
- **Hospitalizations**: Track the frequency of hospitalizations due to infections or other complications. An increase in hospital visits could signal a need for adjustments in treatment (e.g., **immunoglobulin therapy**, **antibiotic prophylaxis**).

---

2. Laboratory Monitoring

Regular **blood tests** and **specialized immune function tests** are essential for assessing the child's immune system and disease progression. These tests help identify deficiencies, monitor treatment efficacy, and catch any emerging issues. Key laboratory tests include:

a. Immunoglobulin Levels

- **IgG, IgA, IgM, and IgE Levels**: Measuring the levels of these antibodies is critical to assess **B-cell function** and determine the need for **immunoglobulin replacement therapy** (e.g., **IVIG** or **SCIG**).
  - **Low IgG levels** indicate a need for **immunoglobulin therapy**.
  - **Normal IgM levels** and **high IgA** may suggest that the B-cells are functioning well, even if IgG is low.

b. Lymphocyte Subsets

- **T-cell (CD4, CD8)** and **B-cell (CD19, CD20)** counts can provide information on the function of the adaptive immune system. **T-cell** and **B-cell deficiencies** can be assessed by analyzing:
  - **CD4/CD8 ratio** (abnormal ratios can suggest **T-cell dysfunction**).
  - **Absolute T-cell count** (low counts indicate **T-cell deficiencies**).
  - **B-cell numbers** (low numbers indicate **B-cell deficiency**, e.g., **XLA**, **CVID**).

c. Specific Antibody Responses

- **Vaccine Response Titers**: After immunization, assess **antibody titers** to specific vaccines (e.g., **pneumococcus**, **tetanus**, **influenza**) to determine if the child is mounting an adequate immune response.
  - **Failure to mount an immune response** to a vaccine may indicate an immune deficiency, especially in the context of **CVID** or other antibody deficiencies.

d. T-cell Function Tests

- **T-cell proliferation assays**: These tests assess how well T-cells respond to mitogens or antigens. **Poor proliferation** may suggest **T-cell deficiencies** (e.g., **SCID**, **DiGeorge syndrome**).
- **Cytokine production assays**: Evaluate how well T-cells produce cytokines (e.g., **IL-2**, **IFN-γ**), which are crucial for immune responses.

e. Neutrophil Function and Phagocytosis

- **Neutrophil counts** and **function tests** like the **NBT (nitroblue tetrazolium) test** or **diisopropylfluorophosphate (DIPF)** test can help assess the function of neutrophils in fighting infections. **Defects in phagocyte function** are common in conditions like **CGD**.
- **Flow cytometry** to evaluate specific markers of phagocytosis or the oxidative burst (important in diseases like **CGD** and **leukocyte adhesion deficiency**).

f. Complement Levels

- **Complement activation and levels** (C3, C4, CH50, C9) should be monitored in children with **complement deficiencies** (e.g., **C3 deficiency** or **properdin deficiency**). Low complement levels can indicate an increased susceptibility to **bacterial infections**.

---

3. Infections Surveillance

Early recognition of infections is critical for children with IEI, as infections may progress quickly. Regular **microbiological surveillance** includes:

- **Blood cultures** to monitor for bacteremia.
- **Chest X-rays** or **CT scans** to check for respiratory infections like **pneumonia**.
- **Sputum cultures** or **nasopharyngeal swabs** to monitor for viral or bacterial infections (especially **respiratory syncytial virus (RSV)**, **influenza**, and **Pneumocystis jirovecii**).
- **Urine cultures** and **stool cultures** to detect urinary tract infections or gastrointestinal infections.
- **Fungal cultures** if a child has symptoms like fever, cough, or skin lesions.

Tracking the frequency of infections over time can help assess the adequacy of **immunoglobulin therapy**, **antibiotic prophylaxis**, and other immune support treatments.

---

4. Immunologic Therapies and Adjustments

As treatment progresses, it is essential to assess the effectiveness of therapies and adjust them as needed:

- **IVIG/SCIG Efficacy**: Monitor how well the child is responding to **immunoglobulin replacement therapy** through clinical improvement and by checking **IgG levels**. Adjust the dose and frequency of therapy based on infection frequency and immunoglobulin levels.
- **Stem Cell Transplant (HSCT)**: After **HSCT**, regular **immune reconstitution assessments** should be done. These include monitoring the recovery of **T-cells**, **B-cells**, and **granulocytes**. Early signs of **graft-versus-host disease (GVHD)** or rejection should be closely monitored.
- **Gene Therapy**: For children receiving **gene therapy**, regular testing of **immune function** (T-cell, B-cell, and antibody responses) is critical to assess if the therapy is providing long-term benefits.

---

5. Assessing Disease Progression

For some **IEI** types, particularly those that involve both immune dysfunction and other organ involvement (e.g., **Ataxia Telangiectasia**, **Wiskott-Aldrich Syndrome**), assessing **disease progression** is essential:

- **Autoimmune Complications**: Monitoring for signs of **autoimmune diseases**, such as **autoantibodies** or **organ inflammation** (e.g., **lupus-like symptoms**, **arthritis**, or **glomerulonephritis**), which are common in **CVID** or **autoimmune-associated IEIs**.
- **Lymphoid Proliferation**: Children with certain **IEIs** (e.g., **CVID**) may develop **lymphoid hyperplasia**, **splenomegaly**, or **lymphomas**, and regular imaging (e.g., **ultrasound**, **CT scans**) may be necessary.

---

6. Genetic Monitoring

For children with genetic diagnoses of **IEI**, assessing **genetic mutations** is important for understanding the disease trajectory and predicting potential complications:

- **Whole-exome sequencing (WES)** or **targeted genetic testing**: This helps identify mutations in the **immune-related genes** that might impact treatment decisions and the likelihood of complications.
- **Genetic counseling**: Involves assessing **inheritance patterns** and advising families about **recurrent risk** for siblings or future children.

---

7. Psychological and Developmental Assessment

Children with IEI may face **psychosocial challenges** due to frequent hospitalizations, infections, and the need for constant monitoring. Developmental milestones should be assessed, and **mental health** support should be integrated into the care plan.

- **Psychological Screening**: Regular screening for **anxiety**, **depression**, and **adjustment disorders** is important for families and patients who may be experiencing stress related to the child's medical condition.

---

8. Multidisciplinary Care

Children with IEI often require a **multidisciplinary care team**, including:

- **Immunologists** to oversee treatment and adjust immune therapy.
- **Infectious disease specialists** for infection prevention and management.
- **Pulmonologists** for respiratory issues.
- **Pediatricians** for general health and growth monitoring.
- **Genetic counselors** for family planning and genetic testing.
- **Psychologists** or **psychiatrists** for addressing mental health concerns.

Conclusion

Regular **clinical monitoring**, **laboratory tests**, and **immunologic assessments** are key to managing children with **inborn errors of immunity (IEI)**. By tracking the child's **immune function**, **treatment responses**, and **infection risk**, healthcare providers can adjust therapies, prevent complications, and improve quality of life. Early intervention and ongoing monitoring are essential for ensuring the best outcomes, as immune deficiencies can evolve over time.

10. What resources and support systems are available for families and caregivers of children with IEI?

Supporting families and caregivers of children with **inborn errors of immunity (IEI)** is crucial for their emotional well-being and the effective management of the child's condition. There are a range of **resources and support systems** available to help families navigate the challenges of managing IEI, both medically and emotionally. Below are some of the most important resources and support systems for families:

---

1. Medical and Clinical Support Networks

a. Immunology and Pediatric Infectious Disease Clinics

- **Specialized Clinics**: Many hospitals and academic medical centers have **immunology clinics** that focus on diagnosing and treating **IEI**. These centers often have multidisciplinary teams that include **immunologists**, **infectious disease specialists**, **nurses**, and **genetic counselors**.
  - These clinics often offer **personalized care plans** and regular follow-ups to ensure optimal management of the child's condition.

b. Pediatric and Genetic Counseling

- **Genetic Counselors**: Families of children with **genetic IEI** (e.g., **SCID**, **Wiskott-Aldrich Syndrome**, **CVID**) can benefit from genetic counseling. These counselors can:
  - Help understand inheritance patterns.
  - Provide information on family planning and recurrence risks.
  - Guide families through genetic testing options.
- **Psychosocial Support**: Families can benefit from **psychosocial support** offered by professionals (psychologists or social workers) who specialize in chronic illness. This includes:
  - **Coping strategies** for stress, anxiety, or grief.
  - **Support for siblings**, who may also experience stress due to the special needs of the affected child.

---

2. Patient Advocacy Groups and Organizations

a. National and International IEI Foundations

- **Immune Deficiency Foundation (IDF)** (U.S.) – **IDF** is one of the leading organizations dedicated to supporting patients and families affected by primary immunodeficiencies. They offer:
    - **Educational materials** on various IEIs.
    - A **helpline** for support and questions.
    - **Patient registries** to connect families.
    - **Advocacy** for better healthcare policies and insurance coverage.
    - **Local support groups** for connecting with other families.
- **European Society for Immunodeficiencies (ESID)** – Offers resources for families in Europe, providing educational materials, information about disease management, and connecting families with other patients and specialists.
- **Pediatric Immune Deficiency Foundation (PIDF)** – A non-profit organization that supports the diagnosis and treatment of children with **primary immunodeficiencies**. PIDF offers:
    - Educational resources for both patients and healthcare providers.
    - Information on how to access genetic testing and therapies.
    - Advocacy for better healthcare policies.
- **The Jeffrey Modell Foundation (JMF)** – Dedicated to increasing awareness, early diagnosis, and improving the quality of life for children with **primary immunodeficiencies**. They provide:
    - Financial assistance for diagnostic testing.
    - Awareness campaigns.
    - Collaboration with hospitals and universities for better research and treatment.

b. Rare Disease Organizations

Many **rare disease** organizations also provide valuable support for children with **IEI**, as many immune deficiencies are considered rare:

- **National Organization for Rare Disorders (NORD)**: A resource for families managing any rare disease, including **IEI**. NORD provides information, research support, and access to financial assistance.
- **Global Genes**: A global organization offering advocacy, educational resources, and a **community network** for families affected by rare diseases.

---

3. Online Communities and Peer Support Groups

a. Online Support Forums and Social Media Groups

- **Facebook Groups**: Many IEI families have created private **Facebook support groups** where they can share experiences, ask questions, and offer advice. Examples of groups include:
    - **Primary Immunodeficiency Parents Support Group**
    - **SCID Awareness and Support Group**
- These groups also provide emotional support, as families can connect with others going through similar experiences.

b. Virtual Support Groups

- Many organizations, like the **Immune Deficiency Foundation (IDF)**, offer virtual **support groups** or **peer-to-peer programs** where families can meet, discuss challenges, and share coping strategies. Some may be specific to **certain IEI types** (e.g., **CVID**, **SCID**).
- **Zoom Calls/Webinars**: Some foundations host **regular webinars** or virtual meetings where experts discuss topics related to **managing IEI**, treatment options, and coping mechanisms.

---

4. Financial and Legal Assistance

a. Insurance and Healthcare Navigation

- **Insurance Assistance**: Families with children who have IEI often face challenges with **insurance coverage** for treatments, therapies, and medications. Many foundations (such as **IDF**) offer **help with insurance advocacy**, providing guidance on how to appeal insurance denials and get **medically necessary treatments** covered.
- **Government Programs**: Families may qualify for government assistance programs such as:
  - **Social Security Disability Insurance (SSDI)** or **Supplemental Security Income (SSI)** for children with disabilities.
  - **Medicaid**: Some states provide Medicaid coverage for children with special healthcare needs.
  - **The Affordable Care Act**: Ensures children with pre-existing conditions (like IEI) cannot be denied insurance coverage.

b. Financial Aid and Grants

- Many IEI-related organizations provide financial support in the form of grants, assistance with **diagnostic testing**, or help paying for **medications**.
  - **The Jeffrey Modell Foundation** offers grants for **diagnostic testing** for families in need.
  - **NORD** also provides **financial assistance** for patients and families dealing with rare diseases.

---

5. Educational Support and School Resources

a. School Re-entry Programs

- **School accommodations**: Children with IEI often face challenges attending school regularly due to infections or hospitalizations. Resources for school accommodations may include:
  - **Homebound education services** provided by the school district.
  - **504 plans** or **Individualized Education Plans (IEPs)** that outline necessary adjustments for **school attendance**, including **medical absences**, **extra time for assignments**, and **infection control measures** in the classroom.

b. Educational Resources for Teachers

- Some foundations, such as the **Immune Deficiency Foundation**, offer resources to help teachers understand IEIs and **special accommodations** for students. These resources can include guidelines on infection control, classroom hygiene, and creating a supportive school environment.

---

6. Mental Health and Counseling Services

a. Mental Health Resources

- **Counseling**: Chronic illness can take an emotional toll on both children and caregivers. Seeking **psychological support** is essential. Support might include:
  - **Therapy** (individual or family counseling).
  - **Support groups** for parents and siblings.
  - **Psychological evaluations** for coping mechanisms, stress management, and anxiety.

b. Hospital-based Support Programs

- Many hospitals with specialized **immunology departments** also have **psychosocial teams** that offer counseling for patients and their families.
  - Some hospitals offer **peer mentorship programs**, where families with children who are newly diagnosed with IEI can receive guidance and emotional support from families with more experience.

---

7. Research and Clinical Trials Information

a. Clinical Trials

- Clinical trials may offer **access to new treatments** for children with IEI. Families can find information about **clinical trials** through:
  - **ClinicalTrials.gov**: A database of global clinical trials.
  - **NORD's Clinical Trial Search Tool**: A resource for finding trials specific to **rare diseases**, including IEIs.

b. Research Advocacy

- Families may also be interested in advocating for more research on IEIs. Organizations like **IDF** often provide ways for families to get involved in **research studies**, support **fundraising for research**, or participate in **patient registries** that help to advance our understanding of IEI.

Conclusion

The emotional and practical challenges of raising a child with **inborn errors of immunity (IEI)** are significant, but families don't have to navigate them alone. There are a wealth of **resources and support systems** available, from **medical care and specialized clinics** to **advocacy groups**, **online support communities**, **financial assistance programs**, and **educational resources**. Accessing these resources can make a huge difference in ensuring the child's health, improving quality of life, and providing the **emotional support** necessary for both the child and the family.

Readability Formulas. *Automated Readability Index (ARI), Flesch Reading Ease Score (FRES), Flesch-Kincaid Grade Level (FKGL) and Gunning Fog Readability Index (GFR)*

ARI = 4.71 × (characters ÷ words) + 0.5 × (words ÷ sentences) - 21.43

FRES = 206.835 - 1.015 (total words ÷ total sentences) - 84.6 (total syllables ÷ total words)

FKGL = 0.39 (total words ÷ total sentences) + 11.8 (total syllables ÷ total words) – 15.49

GFR = 0.4 [(words ÷ sentences) + 100 (complex words ÷ words)]
